# Sensitivity of Optimal Solutions to Control Problems for Second Order Evolution Subdifferential Inclusions

**DOI:** 10.1007/s00245-014-9262-4

**Published:** 2014-07-30

**Authors:** Krzysztof Bartosz, Zdzisław Denkowski, Piotr Kalita

**Affiliations:** Institute of Computer Science, Faculty of Mathematics and Computer Science, Jagiellonian University, ul. Łojasiewicza 6, 30-348 Kraków, Poland

**Keywords:** Evolution subdifferential inclusion, Control problem, Sensitivity, The Clarke subdifferential, Multifunction, Pseudomonotone and maximal monotone operators, $PG$- and $\Gamma$-convergences

## Abstract

In this paper the sensitivity of optimal solutions to control problems described by second order evolution subdifferential inclusions under perturbations of state relations and of cost functionals is investigated. First we establish a new existence result for a class of such inclusions. Then, based on the theory of sequential $\Gamma$-convergence we recall the abstract scheme concerning convergence of minimal values and minimizers. The abstract scheme works provided we can establish two properties: the Kuratowski convergence of solution sets for the state relations and some complementary $\Gamma$-convergence of the cost functionals. Then these two properties are implemented in the considered case.

## Introduction

It is well known ([39, 44–46]) that many problems from mechanics (elasticity theory, semipermeability, electrostatics, hydraulics, fluid flow), economics and so on can be modeled by subdifferential inclusions or hemivariational inequalities. The latter are generalizations of partial differential equations (PDEs) and variational inequalities [26] in the sense that besides of physical phenomena leading to classical PDEs one has to take into consideration some nonlinear, nonmonotone and possibly multivalued laws (e.g. stress–strain, reaction–displacement, generalized forces–velocities, etc.) which can be expressed by means of the Clarke subdifferential.

In this paper, which is in a sense a continuation of [22, 25], we deal with control problems for systems governed by evolution second order inclusions which are equivalent to second order hemivariational inequalities. More precisely, we consider

CP $$\begin{aligned} {\text {minimize}} \ \ \left\{ \mathcal{F}(\mathfrak {u},y) := \mathcal{F}^{(1)}(y) + \mathcal{F}^{(2)}(u) + \mathcal{F}^{(3)}(y_0) + \mathcal{F}^{(4)}(y_1) \right\} \end{aligned}$$

subject to

P $$\begin{aligned} {\left\{ \begin{array}{ll} \displaystyle y''(t) + (\mathcal{A} y')(t)+(\mathcal{B} y)(t) + \iota ^*\partial J^1(\iota y(t))\\ +\iota ^*\partial J^2(\iota y'(t)) \ni f(t) + (C u)(t)\,\,\,\mathrm{{for\, a.e.}}\,t\in (0,T) \\ \displaystyle y(0) = y_0, \ \ \ y'(0)=y_1, \ \ \ y\in \mathcal{V},\ \ \ y' \in \mathcal{W}_{pq}, \ \ \ u\in \mathcal{U}, \\ \end{array}\right. } \end{aligned}$$

where $T>0$, $\mathcal A$ and $\mathcal B$ are the Nemitsky operators corresponding, respectively, to a pseudomonotone operator $A$ and a linear one $B$, $J^1$ and $J^2$ are locally Lipschitz superpotentials defined on a reflexive Banach space $Z$ ($\partial$ denotes their Clarke subdifferentials), $\iota$ is a linear, continuous and compact operator and $C$ is an operator acting on the space $\mathcal{U}$. The control is given as $\mathfrak {u}=(u,y_0,y_1)\in \mathfrak {U}\subset {\mathcal {U}}\times V\times H$, and the cost functionals $\mathcal{F}^{(i)}$, $(i=1,\ldots ,4)$ are typically in integral form (for details and definitions of spaces $V, H$, $\mathcal{V}$ and $\mathcal{W}_{pq}$, see Sect. 3).

Our goal is twofold. First prove a new existence result for the Problem $(P)$ with the sum of two superpotentials, dependent, respectively, on displacement and its velocity. Second we investigate the sensitivity of optimal solutions to the control problem ($CP$); i.e., we are interested in the behavior of optimal solutions under perturbations of the system (state relations; e.g. coefficients in inclusion or parameters in superpotential are perturbed,...) as well as of perturbations of the cost functional (e.g. integrands depending on parameters).

Our approach is based on the sequential $\Gamma$-convergence (epi-convergence in terms of [3]) theory (see [7, 13, 14, 16, 48]) in the sensitivity part, while for the existence of optimal solutions, we use the direct method. The nonemptiness of the solution set for $(P)$ follows from the theory of pseudomonotone operators (cf. [24, 51]) and it can be obtained for fairly general classes of operators. However, for sensitivity results, we restrict ourselves to special classes of maximal monotone operators for which the notion of $PG$-convergence can be applied.

The basic properties assuring the convergence of minimal values and minimizers of perturbed control problems to the minimal value and to a minimizer, respectively, of unperturbed problem are: on one hand the Painlevé-Kuratowski convergence (we use the nomenclature Kuratowski convergence in the sequel, for consistency with our previous works) of solution sets, which can be expressed as $\Gamma$-convergence of their indicator functions and on the other hand some ”complementary $\Gamma$-convergence” of cost functionals.

The sensitivity of control problems was largely considered in the literature in papers on optimal control for systems governed by ordinary differential equations ([7–9, 27]), partial differential equations ([15, 31–34], Chapter 4.2 of [24]), partial differential equations and differential inclusions ([1, 2, 6, 16]). We mention that the related control problems for systems described by Clarke subdifferential inclusions and hemivariational inequalities were studied in [5, 22, 25, 29, 36, 37] the shape optimization problems for Clarke subdifferential inclusions were considered in [18–21, 28, 43] and the corresponding inverse and identification problems were treated in [35]. More recently, the $PG$-convergence approach was used in homogenization problems for initial and boundary value problems for second order (in time) equations with linear damping and nonlinear elliptic terms (the homogenization was done with respect to coefficients present in the elliptic term) by Svanstedt [50], whose work was further extended in [40–42].

The paper is organized as follows. In Sect. 2 we present an abstract setting for the multivalued operators and subdifferential inclusions as well as the sensitivity analysis which is based on the $\Gamma$-convergence theory. Moreover, we recall some useful definitions and results from the theory of Clarke subdifferential and theory of pseudomonotone operators. In Sect. 3 we recall the definition and properties of $PG$ convergence. Next, we present the control problem formulation and provide a priori estimates as well as the existence result for the underlying Problem $(P)$. Furthermore, we analyze the perturbed problems and provide results on the Kuratowski convergence of solution sets, and we formulate sensitivity result. In Sect. 4 we discuss the $\Gamma$-convergence of cost functionals and present the main result on the sensitivity of optimal solutions. In Sect. 5 we give examples of concrete operators and functionals which satisfy the abstract assumptions of preceding sections.

## General Setting and Preliminaries

### Abstract Scheme

In this subsection we recall the abstract scheme based on the $\Gamma$-convergence theory which we use to study the stability of optimal control problems.

We consider a control system governed by a relation $\mathcal{R}$ which links the state $y \in \mathcal{Y}$ to the control variable $\mathfrak {u} \in \mathfrak {U}$, $\mathcal{Y}$ and $\mathfrak {U}$ being the topological spaces of states and controls, respectively. Generally, the relation $\mathcal{R}$ can be chosen as an ordinary differential equation, a partial differential equation or a partial differential inclusion. It is also possible to consider variational inequalities (VI) or hemivariational inequalities (HVI).

The optimal control problem under consideration reads as follows: find $(\mathfrak {u}^*, y^*) \in {\mathcal {R}}$ which minimizes a cost functional $\mathcal{F}$:

$$\begin{aligned} \displaystyle {\text {minimize}} \ \ \left\{ \mathcal{F}(\mathfrak {u},y) \, : \, (\mathfrak {u},y) \in \mathcal{R} \right\} \ \ \ \left( = \mathcal{F}(\mathfrak {u}^*, y^*) = : m \right) ,\quad \quad \quad \quad (CP)_\mathcal{R} \end{aligned}$$

where the set $\mathcal{R}$ of admissible control-state pairs is defined by:

$$\begin{aligned} \displaystyle \mathcal{R} = graph \, \mathcal{S}_\mathcal{R} = \left\{ (\mathfrak {u}, y) \, : \, y \in \mathcal{S}_\mathcal{R}(\mathfrak {u}), \ \ \mathfrak {u} \in \mathfrak {U} \right\} \end{aligned}$$

and the solution map is given by

$$\begin{aligned} \displaystyle \mathcal{S}_\mathcal{R}\, : \, \mathfrak {U} \ni \mathfrak {u} \longrightarrow \mathcal{S}_\mathcal{R}(\mathfrak {u}) = \left\{ y \in \mathcal{Y} \, : \, (\mathfrak {u}, y) \in \mathcal{R} \right\} \subset \mathcal{Y}. \end{aligned}$$

The set of optimal solutions to $(CP)_\mathcal{R}$ is denoted by $\mathcal{R}^*$, i.e.,

$$\begin{aligned} \displaystyle \mathcal{R}^* = \left\{ (\mathfrak {u}^*, y^*) \in \mathcal{R} \, : \, \mathcal{F}(\mathfrak {u}^*, y^*) = m \right\} . \end{aligned}$$

The sensitivity (stability) is understood as a “nice-continuous” asymptotic behavior of optimal solutions to the perturbed problems, i.e. perturbed state relations $\mathcal{R}_k$ and perturbed cost functionals $\mathcal{F}_k$. So we consider the sequence of optimal control problems indexed by $k \in {\bar{\mathbb {N}}} = \mathbb {N}\cup \{ \infty \}$, where the index $k \in \mathbb {N}$ indicates “a perturbation” and $k = \infty$ corresponds to the unperturbed original problem:

$$\begin{aligned} \displaystyle {\text {minimize}} \ \ \left\{ \mathcal{F}_k (\mathfrak {u},y) \, : \, (\mathfrak {u},y) \in \mathcal{R}_k \right\} \ \ \ \left( = \mathcal{F}_k (\mathfrak {u}_k^*, y_k^*) =: m_k \right) \quad \quad \quad (CP)_{\mathcal{R}_k} \end{aligned}$$

and $\mathcal{R}_k = graph \, \mathcal{S}_{\mathcal{R}_k}$. We are looking for conditions which assure the following stability results:

(i)       $$m_k \rightarrow m_\infty $$ as $$k \rightarrow \infty $$,
(ii)       $$K(\mathfrak {U}\times {\mathcal {Y}})- \limsup \mathcal{R}_k^* \subset \mathcal{R}^*_\infty $$,

where $K(\mathfrak {U}\times {\mathcal {Y}})- \limsup$ stands for the sequential Kuratowski upper limit of sets. It is worth to recall (see e.g. Proposition 4.3 of [16]) that (ii) is equivalent to the following condition: if $\{ k_n \}$ is an increasing sequence in $\mathbb {N}$, $(\mathfrak {u}^*_{k_n}, y^*_{k_n}) \in \mathcal{R}_{k_n}^*$, $\mathfrak {u}^*_{k_n}$ converges to $\mathfrak {u}^*_{\infty }$ in $\mathfrak {U}$ and $y^*_{k_n}$ converges to $y^*_{\infty }$ in $\mathcal{Y}$, then $(\mathfrak {u}^*_{\infty }, y^*_{\infty }) \in \mathcal{R}_{\infty }^*$.

In order to establish the conditions (i) and (ii), first we reformulate the problem $(CP)_{\mathcal{R}_k}$ as the unconstrained optimization one:

$$\begin{aligned} \displaystyle {\text {minimize}} \ \ \left\{ \mathcal{F}_k (\mathfrak {u},y) + \delta _{\mathcal{R}_k}(\mathfrak {u},y) \, : \, (\mathfrak {u},y) \in \mathfrak {U} \times \mathcal{Y} \right\} , \quad \quad \quad (CP)_{\mathcal{R}_k} \end{aligned}$$

where $\delta _\mathcal{R}$ denotes the indicator function of the set $\mathcal{R}$, i.e.,

$$\begin{aligned} \delta _\mathcal{R}(x) = {\left\{ \begin{array}{ll} 0 &{} x \in \mathcal{R} \\ +\infty &{} x \notin \mathcal{R} , \\ \end{array}\right. } \end{aligned}$$

and then we apply an approach based on the theory of $\Gamma$-convergence (epi-convergence), cf. [7, 13, 48], and the references therein.

### Sequential $\Gamma$-convergence

For the convenience of the reader in this subsection we recall some material from the $\Gamma$-convergence theory, the generalized Clarke subdifferential and the theory of multivalued operators of monotone type.

We quote here the definition of $\Gamma _{seq}$-convergence for functions of two variables. The case of one variable follows easily by omitting the other. For the case of functions of many variables we refer to Buttazzo and Dal Maso [7].

Let $\mathfrak {U}$ and $\mathcal{Y}$ be two topological spaces. For $\mathfrak {u} \in \mathfrak {U}$ and $y \in \mathcal{Y}$ we put $\displaystyle \sigma _\mathfrak {u} := \{ \{ \mathfrak {u}_k \} \subset \mathfrak {U} \, : \, \mathfrak {u}_k \rightarrow \mathfrak {u} \}$ and $\displaystyle \sigma _y := \{ \{ y_k \} \subset \mathcal{Y} \, : \, y_k \rightarrow y \}$. Given $\mathcal{F}_k :\mathfrak {U} \times \mathcal{Y} \rightarrow {\bar{\mathbb {R}}} = {\mathbb {R}} \cup \{ \pm \infty \}$, $k \in \mathbb {N}$, we define

$$\begin{aligned}&\displaystyle \Gamma _{seq}(\mathfrak {U}^{-}, \mathcal{Y}^{+}) \liminf _{k\rightarrow \infty } \mathcal{F}_k(\mathfrak {u}, y) = \inf _{\sigma _\mathfrak {u}} \ \sup _{\sigma _y} \ \liminf _{k\rightarrow \infty } \mathcal{F}_k(\mathfrak {u}_k, y_k),\\&\displaystyle \Gamma _{seq}(\mathfrak {U}^{-}, \mathcal{Y}^{+}) \limsup _{k\rightarrow \infty } \mathcal{F}_k(\mathfrak {u}, y) = \inf _{\sigma _\mathfrak {u}} \ \sup _{\sigma _y} \ \limsup _{k\rightarrow \infty } \mathcal{F}_k(\mathfrak {u}_k, y_k), \end{aligned}$$

and if both these extended real numbers are equal, we say that there exists

j $$\begin{aligned} \displaystyle \Gamma _{seq}(\mathfrak {U}^{-}, \mathcal{Y}^{+}) \lim _{k\rightarrow \infty } \mathcal{F}_k(\mathfrak {u}, y). \end{aligned}$$

Similarly, for other combination of signs ($+$ and $-$ denote $\sup$ and $\inf$, respectively) we have

$$\begin{aligned}&\displaystyle \Gamma _{seq}(\mathfrak {U}^{-}, \mathcal{Y}^{-}) \liminf _{k\rightarrow \infty } \mathcal{F}_k(\mathfrak {u}, y) = \inf _{\sigma _\mathfrak {u}} \ \inf _{\sigma _y} \ \liminf _{k\rightarrow \infty } \mathcal{F}_k(\mathfrak {u}_k, y_k),\\&\displaystyle \Gamma _{seq}(\mathfrak {U}^{-}, \mathcal{Y}^{-}) \limsup _{k\rightarrow \infty } \mathcal{F}_k(\mathfrak {u}, y) = \inf _{\sigma _\mathfrak {u}} \ \inf _{\sigma _y} \ \limsup _{k\rightarrow \infty } \mathcal{F}_k(\mathfrak {u}_k, y_k), \end{aligned}$$

and if they are equal there exists

jj $$\begin{aligned} \displaystyle \Gamma _{seq}(\mathfrak {U}^{-}, \mathcal{Y}^{-}) \lim _{k\rightarrow \infty } \mathcal{F}_k(\mathfrak {u}, y). \end{aligned}$$

In turn, if the numbers in $(j)$ and $(jj)$ are equal, we say that there exists

$$\begin{aligned} \displaystyle \Gamma _{seq}(\mathfrak {U}^{-}, \mathcal{Y}^{\pm }) \lim _{k\rightarrow \infty } \mathcal{F}_k(\mathfrak {u}, y) \end{aligned}$$

and then we write simply

$$\begin{aligned} \displaystyle \Gamma _{seq}(\mathfrak {U}^{-}, \mathcal{Y}) \lim _{k\rightarrow \infty } \mathcal{F}_k(\mathfrak {u}, y) = (j) = (jj). \end{aligned}$$

The general definition of a topological $\Gamma -$limit is given by De Giorgi and Franzoni in [14], where one can also find the following theorem concerning the variational convergence of minimal values and minimizers.

#### **Theorem 1**

Let $X$ be a topological space and let $\displaystyle f_k :X \rightarrow {\bar{\mathbb {R}}} = \mathbb {R}\cup \{ \pm \infty \}$, $k \in {\bar{\mathbb {N}}}$ be such that $\displaystyle f_\infty = \Gamma (X^{-}) \lim _{k\rightarrow \infty } f_k$. If

$$\begin{aligned} \liminf _{k\rightarrow \infty } f_k ({\widehat{x}}_k) = \liminf _{k\rightarrow \infty } \left( \inf _X f_k(x) \right) \end{aligned}$$

(in this case ${\widehat{x}}_k$ is called to be “quasioptimal”) and

$$\begin{aligned} {\widehat{x}}_{k_n} \rightarrow {\widehat{x}}_\infty \ \ \mathrm{as} \ \ n \rightarrow \infty , \end{aligned}$$

then $$\displaystyle f_\infty ({\widehat{x}}_\infty ) = \inf _X f_\infty (x) = \lim _{k\rightarrow \infty } f_k ({\widehat{x}}_k)$$.

In the sequel we put

$$\begin{aligned}&\displaystyle X = \mathfrak {U} \times \mathcal{Y}, \ \ \ \mathcal{R}_k = (P)_k\\&\displaystyle \mathcal{S}_k = \mathcal{S}_{\mathcal{R}_k} \ \ {\text {and}} \ \ f_k(x) = \mathcal{F}_k(\mathfrak {u},y) + \delta _{\mathcal{R}_k}(\mathfrak {u},y). \end{aligned}$$

#### *Remark 2*

If the topological space $X$ satisfies the first axiom of countability, then the sequential $\Gamma _{seq}(X^{-})$-convergence coincides (see Proposition 8.1 of [12]) with the topological $\Gamma (X^{-})$-convergence of De Giorgi and Franzoni [14]. Moreover, the sequential $\Gamma$-limit operation is not additive, i.e. it is not enough to know $\Gamma \lim \mathcal{F}_k$ and $\Gamma \lim \delta _{\mathcal{R}_k}$ in order to calculate $\Gamma \lim (\mathcal{F}_k + \delta _{\mathcal{R}_k})$, cf. Example 6.18 in [12].

In order to calculate the $\Gamma$-limit of the sum of two functions we use the following two theorems

#### **Theorem 3**

(Buttazzo and Dal Maso [7]) If

$$\begin{aligned}&\displaystyle \mathcal{F}(\mathfrak {u},y) = \Gamma _{seq}(\mathfrak {U}^{-}, \mathcal{Y}) \lim _{k\rightarrow \infty } \mathcal{F}_k(\mathfrak {u}, y),\\&\displaystyle \mathcal{G}(\mathfrak {u},y) = \Gamma _{seq}(\mathfrak {U}, \mathcal{Y}^{-}) \lim _{k\rightarrow \infty } \mathcal{G}_k(\mathfrak {u}, y), \end{aligned}$$

then

$$\begin{aligned} \displaystyle \mathcal{F}(\mathfrak {u},y) + \mathcal{G}(\mathfrak {u}, y) = \Gamma _{seq}(\mathfrak {U}^{-}, \mathcal{Y}^{-}) \lim _{k\rightarrow \infty } \left( \mathcal{F}_k(\mathfrak {u}, y) + \mathcal{G}_k(\mathfrak {u}, y) \right) . \end{aligned}$$

#### **Theorem 4**

If there exist

$$\begin{aligned}\mathcal{F}^{(1)}(x)=\Gamma _{seq}(\mathcal{X}^{-})\lim _{k\rightarrow \infty }\mathcal{F}^{(1)}_k(x)\end{aligned}$$

and

$$\begin{aligned}\mathcal{F}^{(2)}(y)=\Gamma _{seq}(\mathcal{Y}^{-})\lim _{k\rightarrow \infty }\mathcal{F}^{(2)}_k(y),\end{aligned}$$

then there exists also

$$\begin{aligned}\Gamma _{seq}((\mathcal{X}\times \mathcal{Y} )^{-})\lim _{k\rightarrow \infty }[\mathcal{F}^{(1)}_k(x)+\mathcal{F}^{(2)}_k(y)]\end{aligned}$$

and we have

$$\begin{aligned} \Gamma _{seq}((\mathcal{X}\times \mathcal{Y} )^{-})\lim _{k\rightarrow \infty }[\mathcal{F}^{(1)}_k(x)+\mathcal{F}^{(2)}_k(y)]=\mathcal{F}^{(1)}(x)+\mathcal{F}^{(2)}(y). \end{aligned}$$

Note that Theorem 4 follows directly from Theorem 3. Moreover, due to Theorem 3, the convergences

(i)       $m_k \rightarrow m_\infty$ (of minimal values) and
(ii)       $$K(\mathfrak {U}\times {\mathcal {Y}})- \limsup \mathcal{R}_k^* \subset \mathcal{R}_\infty ^*$$,

follow from the following result (see also Propositions 4.1 and 4.5 in [16]):

#### **Proposition 5**

Suppose

1 $$\begin{aligned} \mathcal{F}_\infty = \Gamma _{seq}(\mathfrak {U}^{-}, \mathcal{Y}) \lim \mathcal{F}_k, \end{aligned}$$

2 $$\begin{aligned} \delta _{{\mathcal {R}}_\infty } = \Gamma _{seq}(\mathfrak {U}, \mathcal{Y}^{-}) \lim \delta _{{\mathcal {R}}_k}. \end{aligned}$$

Let $({\widehat{\mathfrak {u}}}_k, {\widehat{y}}_k)$ be optimal or “quasioptimal solutions” to the problems $(CP)_{\mathcal{R}_k}$ such that

3 $$\begin{aligned} \liminf _{k\rightarrow \infty } \mathcal{F}_k({\widehat{\mathfrak {u}}}_k, {\widehat{y}}_k) = \liminf _{k\rightarrow \infty } \left( \inf _{\mathcal{R}_k} \mathcal{F}_k \right) \end{aligned}$$

and

4 $$\begin{aligned} ({\widehat{\mathfrak {u}}}_{k_n}, {\widehat{y}}_{k_n}) \rightarrow ({\widehat{\mathfrak {u}}}_\infty , {\widehat{y}}_\infty ) \ {\text {as}} \ n \rightarrow \infty . \end{aligned}$$

Then $$\displaystyle \mathcal{F}_\infty ({\widehat{\mathfrak {u}}}_\infty , {\widehat{y}}_\infty ) = \inf _{\mathcal{R}_\infty } \mathcal{F}_\infty (\mathfrak {u}, y) = \lim _{k\rightarrow \infty } \left( \inf _{\mathcal{R}_k} \mathcal{F}_k (\mathfrak {u}, y) \right) $$.

#### *Remark 6*

The condition (2) of Proposition 5 is equivalent (cf. Propositions 4.3 and 4.4 of [16]) to the sequential Kuratowski convergence

2' $$\begin{aligned} \displaystyle \mathcal{S}_k (\mathfrak {u}_k) \ \ \mathop {\longrightarrow }\limits ^{K({\mathcal{Y}})} \ \ \mathcal{S}_\infty (\mathfrak {u}) \ \ \mathrm{{for\, all}} \ \ \mathfrak {u}_k \ \mathop {\longrightarrow }\limits ^{\mathfrak {U}} \ \mathfrak {u}, \end{aligned}$$

i.e.

2'' $$\begin{aligned} \displaystyle K(\mathcal{Y})-\limsup \mathcal{S}_k (\mathfrak {u}_k) \subset \mathcal{S}_\infty (\mathfrak {u}) \subset K(\mathcal{Y})-\liminf \mathcal{S}_k (\mathfrak {u}_k) \ \ \mathrm{{for\, all}} \ \ \mathfrak {u}_k \ \mathop {\longrightarrow }\limits ^{\mathfrak {U}} \ u, \end{aligned}$$

while the condition (1) (the complementary $\Gamma$-convergence), roughly speaking, means a continuous convergence of cost functionals with respect to $y$ and $\Gamma (\mathfrak {U}^-)$ convergence with respect to $\mathfrak {u}$. Note that for the sequence of operators $G_k:{\mathcal {X}}\rightarrow {\mathcal {Y}}$, where ${\mathcal {X}}$ and ${\mathcal {Y}}$ are topological spaces, we say that $G_k$ converges continuously (sequentially) to $G_\infty$ ($G_k\mathop {\longrightarrow }\limits ^{c}G_\infty$) if for every sequence $x_k\rightarrow x_\infty$, we have $G_k(x_k)\rightarrow G_\infty (x_\infty )$. We also recall that for a sequence of sets $\{ A_n \}_{n\in \mathbb {N}}$ in the topological space ${\mathcal {X}}$, by $K({\mathcal {X}})-\liminf A_n$ we mean the set of all limits of sequences $\{ x_n \}$ such that $x_n \in A_n$, while the set $K({\mathcal {X}})-\limsup A_n$ consists of all limits of subsequences $\{ x_k \}$ such that $x_k \in A_{n_k}$ for any increasing sequence $\{ n_k \} \subset \{ n \}$.

### Clarke Subdifferential

Given a locally Lipschitz function $J :Z \rightarrow \mathbb {R}$, where $Z$ is a Banach space, we recall (see [10]) the definitions of the generalized directional derivative and the generalized gradient of Clarke. The generalized directional derivative of $J$ at a point $u \in Z$ in the direction $v \in Z$, denoted by $J^{0}(u;v)$, is defined by

$$\begin{aligned}\displaystyle J^{0}(u; v) = \limsup _{y \rightarrow u, \ t\downarrow 0} \frac{J(y + t v) - J(y)}{t}. \end{aligned}$$

The generalized gradient of $J$ at $u$, denoted by $\partial J(u)$, is a subset of a dual space $Z^*$ given by $\partial J(u) = \{ \zeta \in Z^* : J^{0}(u; v) \ge {\langle \zeta , v \rangle }_{Z^* \times Z}$ for all $v \in Z \}$. For the properties of Clarke subdifferential, see for example [10].

### Multivalued Operators

We give the basic definitions for multivalued operators and then we quote the main surjectivity result for the operator classes under consideration (see e.g. [24, 39, 47]). Let $Y$ be a reflexive Banach space and $Y^*$ be its dual space and let $\displaystyle T :Y \rightarrow 2^{Y^*}$ be a multivalued operator.

We say that $T$ is:

1. upper semicontinuous if for any closed subset $C \subseteq Y^*$, the set $T^-(C)=\{y \in Y \, : \, Ty \cap C \ne \emptyset \}$ is closed in $Y$, Let $L :D(L) \subset Y \rightarrow Y^*$ be a linear, densely defined and maximal monotone operator.
2. $T$ is $L$-generalized pseudomonotone, if the following conditions hold:
  - for every $y\in Y$, $Ty$ is a nonempty, convex and weakly compact subset of $Y^*$,
  - $T$ is upper semicontinuous from each finite-dimensional subspace of $Y$ into $Y^*$ equipped with the weak topology,
  - if $\{y_n\}\subseteq D(L),\ y_n\longrightarrow y$ weakly in $Y$, $y\in D(L)$, $Ly_n\longrightarrow Ly$ weakly in $Y^*$, $y_n^*\in Ty_n$, $y_n^*\longrightarrow y^*$ weakly in $Y^*$ and $\displaystyle \limsup _{n\rightarrow +\infty }\langle y_n^*,y_n-y \rangle \le 0$, then $y^*\in Ty$ and $\langle y_n^*,y_n \rangle \longrightarrow \langle y^*,y \rangle$.

The crucial point in the proof of the existence of a solution to the subdifferential inclusions considered below is the following surjectivity result.

#### **Proposition 7**

If $Y$ is a reflexive, strictly convex Banach space, $L :D(L) \subset Y \rightarrow Y^*$ is a linear, densely defined, maximal monotone operator and $\displaystyle T :Y \rightarrow 2^{Y^*}\setminus \{\emptyset \}$ is a bounded, coercive and $L$-generalized pseudomonotone operator, then $L + T$ is surjective.

The proof of Proposition 7 can be found in [47], Theorem 2.1, p. 345.

## Control Problem for Second Order Subdifferential Inclusion

In this section we consider optimal control problem for systems described by evolution of second order subdifferential inclusion. We first recall the notion of parabolic $G$-convergence ($PG$-convergence) of operators, then we state a result on the sensitivity of the solution set.

### Notation

Let $\Omega$ be an open bounded subset of ${\mathbb {R}}^{N}$ and let $V = W^{1,p}_0(\Omega )$, $H = L^{2}(\Omega )$, $V^* = W^{-1,q}(\Omega )$, where $2 \le p < \infty$ and $1/p + 1/q = 1$. Moreover, we consider a reflexive and separable Banach space $Z$ and a linear continuous and compact mapping $\iota : V\rightarrow Z$. Then $V \subset H \subset V^*$ with compact embeddings. We denote, respectively, by $\langle \cdot ,\cdot \rangle$ and $(\cdot ,\cdot )$ the duality between $V$ and its dual $V^*$ and the inner product in $H$, and by $\Vert \cdot \Vert$, $|\cdot |$, $\Vert \cdot \Vert _{V^*}$ the norms in $V$, $H$ and $V^*$, respectively. Moreover, the adjoint operator to $\iota$ is denoted by $\iota ^*:Z^*\rightarrow V^*$. Given $0 < T < +\infty$, let $Q = \Omega \times (0, T)$. We introduce the following spaces $\displaystyle \mathcal{V} = L^{p}(0,T;V)$, $\displaystyle \mathcal{Z} = L^{p}(0,T;Z)$, $\displaystyle \mathcal{H} = L^{2}(0,T;H) \simeq L^2(Q)$, $\displaystyle \mathcal{Z^*} = L^{q}(0,T;Z^*)$, $\displaystyle \mathcal{V^*} = L^{q}(0,T;V^*)$ and $\displaystyle \mathcal{W}_{pq} = \{ v \in \mathcal{V} \, : \, v' \in \mathcal{V^*} \}$. The duality for the pair $({\mathcal {V}},{\mathcal {V}}^*)$ is denoted by $\langle \langle f,v \rangle \rangle _{{{\mathcal {V}}}^*\times {\mathcal {V}}}=\int _0^T\langle f(t),v(t) \rangle dt$. It is well known [51] that

$$\begin{aligned} \displaystyle \mathcal{W}_{pq} \subset \mathcal{V} \subset \mathcal{H} \subset \mathcal{V}^{*}, \end{aligned}$$

and $\mathcal{W}_{pq}$ is embedded in $C(0,T;H)$ continuously. We set $\mathcal{Y}=\{v \in \mathcal{V}\, :\, v'\in \mathcal{W}_{pq}\}$. The space $\mathcal{Y}$ is endowed with the topology $\Xi$ defined in the following wayWe assume that the Nemytskii operator ${\bar{\iota }}:{\mathcal {W}}_{pq}\rightarrow {\mathcal {Z}}$ corresponding to $\iota$, is compact (for simplicity in the sequel we use the same symbol $\iota$ for its Nemytskii operator). For example, in particular application, we put $Z=L^p(\Omega )$ and $\iota$ being the embedding operator. Then, by the Lions–Aubin Lemma, we know that required compactness of ${\bar{\iota }}$ holds. Note, moreover, that if $v_n{\buildrel {\Xi } \over \longrightarrow } v$, then, by the Lions–Aubin Lemma, $v_n\rightarrow v$ and $v_n'\rightarrow v'$ strongly in ${\mathcal {H}}$. Moreover, if $v_n{\buildrel {\Xi } \over \longrightarrow } v$, then $v_n(t)\rightarrow v(t)$ weakly in $V$ and $v_n'(t)\rightarrow v'(t)$ weakly in $H$ for all $t\in [0,T]$.

### PG-Convergence of Parabolic Operators

Following Svanstedt [49] we start with the following definition.

#### **Definition 8**

Given nonnegative constants $m_0$, $m_1$, $m_2$ and $0 < \alpha \le 1$, we set

$$\begin{aligned} \displaystyle \mathcal{M} = \mathcal{M}(m_0, m_1, m_2, \alpha ) := \{ a :Q \times {\mathbb {R}}^N \rightarrow {\mathbb {R}}^N \ \mathrm{such \ that} \ {(i)-(iv)} \ \mathrm{below \ hold} \} \end{aligned}$$

(i) $$|a(t, x, 0)| \le m_0$$ a.e.in $$Q$$;
(ii) $a(\cdot ,\cdot , \xi )$ is Lebesgue measurable on $Q$ for all $\xi \in {\mathbb {R}}^N$;
(iii) $\displaystyle |a(t,x,\xi ) - a(t,x,\eta )| \le m_1 ( 1 + |\xi | + |\eta |)^{p-1-\alpha }|\xi - \eta |^\alpha$ a.e. in $Q$ , for all $\xi$, $\eta$;
(iv) $\displaystyle (a(t,x,\xi ) - a(t,x,\eta ), \xi - \eta )_{{\mathbb {R}}^N} \ge m_2 |\xi - \eta |^\alpha$ a.e. in $Q$, for all $\xi$, $\eta \in {\mathbb {R}}^N$.

#### *Remark 9*

If $\displaystyle a \in \mathcal{M}$, then the following inequalities hold

$$\begin{aligned} {\left\{ \begin{array}{ll} \displaystyle |a(t,x,\xi )| \le c_1 (1 + |\xi |)^{p-1} \ \mathrm{{a.e.\,in}} \ Q, \ \mathrm{{for\,all}} \ \xi \in {\mathbb {R}}^N \\ \displaystyle |\xi |^p \le c_2 (1 + (a(t,x,\xi ), \xi )_{{\mathbb {R}}^N}) \ \mathrm{{a.e.\,in}} \ Q, \ \mathrm{{for\,all}} \ \xi \in {\mathbb {R}}^N \\ \end{array}\right. } \end{aligned}$$

so the mappings from the class $\mathcal{M}$ are uniformly bounded, coercive and monotone.

#### **Definition 10**

A sequence of maps $a_k \in \mathcal{M}$ is $PG$ convergent to a map

$a_\infty \in \mathcal{M}$, written as $\displaystyle a_k \ {\buildrel PG \over \longrightarrow } \ a_\infty$, if for every $g \in \mathcal{V^*}$ we have

$$\begin{aligned} {\left\{ \begin{array}{ll} \displaystyle y_k \ {\buildrel w-\mathcal{W}_{pq} \over \longrightarrow } \ y_\infty , \\ a_k(t,x,Dy_k) \ \ {\buildrel w-L^q(Q,{\mathbb {R}}^N) \over \longrightarrow } \ \ a_\infty (t,x, Dy_\infty ), \\ \end{array}\right. } \end{aligned}$$

where $y_k$, $k \in {\bar{\mathbb {N}}} = \mathbb {N}\cup \{ \infty \}$, is the unique solution to the problem

5 $$\begin{aligned} y' - \text {div} a_k(t,x,Dy) = g, \ \ y(0) = 0. \end{aligned}$$

Here and in the sequel the symbol $D$ denotes the gradient operator taken with respect to the space variable $x\in \Omega$ and the symbol $\text {div}$ denotes the divergence with respect to the space variable $x\in \Omega$.

#### *Remark 11*

Given $a_k \in \mathcal{M}$, it can be shown that the Nemitsky operators $\mathcal{A}_k :\mathcal{V} \rightarrow \mathcal{V^*}$ of the form

$$\begin{aligned} (\mathcal{A}_k y)(t) = A_k (t,y), \ \ t \in (0, T) \end{aligned}$$

corresponding to the family of operators $A_k (t,y) = - \,\mathrm{div}\, a_k(t,x,Dy)$ are bounded, coercive, hemicontinuous and monotone. Therefore, by Proposition 7, for every $k \in {\bar{\mathbb {N}}}$ and $g \in \mathcal{V^*}$, there exists a unique solution $y_k \in \mathcal{W}_{pq}$ to the problem (5). The compactness of the class $\mathcal{M}$ with respect to the $PG$-convergence was established in [49]. The Definition 10 generalizes the one given for a class of linear operators by Colombini and Spagnolo in [11].

#### *Remark 12*

We use the notion of **parabolic** convergence to deal with the **second order** (in time) problem. This approach is possible due to the fact that the viscosity operator is coercive and hence the nature of the problem is parabolic. It remains an open problem, whether Definition 10 can be modified to include the second time derivative in the auxiliary problem (5). This would require to show the compactness of the underlying class of operators with respect to this new mode of convergence.

### Problem Statement

We consider the following sequence of second order subdifferential inclusions:

$$\begin{aligned} {\left\{ \begin{array}{ll} y''(t) + A_k(t, y'(t)) + B_k y(t) \\ +\iota ^* \partial J^1_k (\iota y'(t)) + \iota ^* \partial J^2_k (\iota y(t)) \ni f_k(t) + (C_k u)(t) \ \ \text {a.e.}\, t\in (0,T)\\ y(0) = y_k^0,\,\, y'(0)=y_k^1,\ \ y\in \mathcal{Y}, \end{array}\right. }\quad \quad (P)_k \end{aligned}$$

for $k \in {\bar{\mathbb {N}}}$, where $J^1_k :Z \rightarrow {\mathbb {R}}$ and $J^2_k :Z \rightarrow {\mathbb {R}}$ are superpotentials. Furthermore $f_k \in \mathcal{V^*}$, $C_k :\mathcal{U} \rightarrow \mathcal{V^*}$, $y_k^0 \in V$ and $y_k^1\in H$.

The hypotheses on the data of $(P)_k$ are the following.

$\underline{H(A)}: \quad \displaystyle A_k :(0, T) \times V \rightarrow V^*$ are the operators of the form

   $$A_k (t, y) = - \text {div}\, a_k(t,x,Dy)$$ with $$a_k \in \mathcal{M}$$, $$k \in {\bar{\mathbb {N}}}$$ and $$\displaystyle a_k \ {\buildrel PG \over \longrightarrow } \ a_\infty $$.

$\underline{H(B)}: \quad \displaystyle B_k \in {\mathcal {L}}(V;V^*)$ is the family of operators such that

(i) $\langle B_k y,v\rangle \le M \Vert y\Vert \Vert v||$, $\langle B_k y,y\rangle \ge 0$ for all $y,v\in V$, $k\in \bar{\mathbb {N}}$ with $M>0$;
(ii) the sequence ${\mathcal {B}}_k:{\mathcal {V}}\rightarrow {\mathcal {V}}^*$ of Nemytski operators corresponding to $B_k$ defined by $({\mathcal {B}}_ky)(t)=B_ky(t)$ satisfies
  $$\begin{aligned} \text {if}\,\,y_k{\buildrel {\Xi } \over \longrightarrow } y_\infty \,\,\,\text {then}\,\,\,{\mathcal {B}}_ky_k\rightarrow {\mathcal {B}}_\infty y_\infty \,\,\,\text {strongly}\,\,\,\text {in}\,\,\,{\mathcal {V}}^*. \end{aligned}$$

$\underline{H(J^1)}: \quad \displaystyle J^1_k :Z \rightarrow {\mathbb {R}}$ are locally Lipschitz functions that satisfy

uniformly in $k$ the conditions

(i) $\displaystyle ||\partial J^1_k(z)||_{Z^*} \le c_3 (1 + ||z||_Z^{p-1})$ for all $z \in Z$ with some $c_3 > 0$;
(ii) $\inf _{\xi \in \partial J^1_k(z)}\langle \xi ,z \rangle _{{Z}^*\times Z} \ge c_4 - c_5 \Vert z \Vert _Z^p$ for all $z\in Z$ with $c_4\in \mathbb {R}$ and $c_5 \ge 0$;
(iii) $$\displaystyle K(s-Z, w-Z^*) -\limsup _{k\rightarrow \infty } Gr \, \partial J^1_k \subset Gr \, \partial J^1_\infty $$.

$\underline{H(J^2)}: \quad \displaystyle J^2_k :Z \rightarrow {\mathbb {R}}$ are locally Lipschitz functions that satisfy

uniformly in $k$ the conditions

(i) $\displaystyle ||\partial J^2_k(z)||_{Z^*} \le c_6 (1 + ||z||_Z^{p-1})$ for all $z \in Z$ with some $c_6 > 0$;
(ii) $$\displaystyle K(s-Z, w-Z^*) -\limsup _{k\rightarrow \infty } Gr \, \partial J_k^2 \subset Gr$$ $$\partial J_\infty ^2$$.

$\underline{H(C)}: \quad C_k \in \mathcal{L}(\mathcal{U}, \mathcal{V}^*)$ and $\Vert C_k\Vert _{{\mathcal {L}}({\mathcal {U}};{\mathcal {V}}^*)}$ are bounded uniformly for $k \in {\bar{\mathbb {N}}}$, where $\mathcal{U}$ is a reflexive separable Banach space and $\displaystyle C_k \ {\buildrel c \over \longrightarrow } \ C_\infty$ continuously.

$$\underline{(H_0)}: \quad y_k^0 \in V$$, $$y_k^1\in H$$, $$f_k \in \mathcal{V}^*$$, $$k \in {\bar{\mathbb {N}}}$$, $$y_k^0\ {\buildrel s-V \over \longrightarrow } y^0_\infty $$, $$y_k^1 \ {\buildrel s-H \over \longrightarrow } \ y^1_\infty $$ , $$f_k \ {\buildrel s-\mathcal{V^*} \over \longrightarrow } \ f_\infty $$.

$\underline{(H_1)}$ : The following relation holds

$$\begin{aligned} \Vert \iota \Vert ^p\left( c_5+c_6T^{p-1}\right) <\frac{1}{c_2}. \end{aligned}$$

We start with a priori estimate for the solution of the problem $(P)_k$. To this end, we give the following lemma.

#### **Lemma 13**

If the assumptions ${H(A)}$, ${H(B)}\mathrm{(i)}$, ${H(J^1)}$, ${H(J^2)}$, ${(H_0)}$, ${H(C)}$ and ${(H_1)}$ hold and $y$ is a solution of the problem $(P)_k$, then it satisfies

$$\begin{aligned} \Vert y\Vert _{C(0,T;V)}^p +\Vert y'\Vert ^2_{C(0,T;H)}+\Vert y'\Vert ^p_\mathcal{V}+\Vert y''\Vert ^q_\mathcal{V^*} \end{aligned}$$

6 $$\begin{aligned} \le C(1+\Vert y^0_k\Vert ^p+|y^1_k|^2+\Vert C_k\Vert ^q_{\mathcal{L}(\mathcal{U}; \mathcal{V}^*) }\Vert u\Vert ^q_\mathcal{U}+\Vert f_k\Vert _\mathcal{V^* }^q), \end{aligned}$$

with a constant $C>0$ dependent only on $T, \Omega$ and the constants $M, c_i$, $i=1,\ldots ,6$.

#### *Proof*

Let $y\in \mathcal{Y}$ be a solution of the problem $(P)_k$. Taking the duality brackets with $y'(t)\in V$ and integrating over $(0,t)$ for any $t\in (0,T)$, we have

7 $$\begin{aligned}&\displaystyle \int \limits _0^t\langle y''(s),y'(s) \rangle \,ds+\int \limits _0^t\langle A_k(s,y'(s)),y'(s) \rangle \,ds+\int \limits _0^t\langle B_k y(s),y'(s) \rangle \,ds\nonumber \\&\displaystyle +\int \limits _0^t\langle \xi (s),\iota y'(s) \rangle _{{Z}^*\times Z}\,ds+\int \limits _0^t\langle \zeta (s),\iota y'(s) \rangle _{{Z}^*\times Z}\,ds\nonumber \\&\displaystyle =\int \limits _0^t\langle f_k(s),y'(s) \rangle \,ds+\int \limits _0^t\langle (C_ku)(s),y'(s) \rangle \,ds \end{aligned}$$

with $\xi (s)\in \partial J^1_k(\iota y'(s))$ and $\zeta (s)\in \partial J^2_k(\iota y(s))$ for a.e. $s\in (0,t)$. From the integration by parts formula (Proposition 23.23(iv), pp. 422–423 of [51]), we get

8 $$\begin{aligned} \int \limits _0^t\langle y''(s),y'(s) \rangle \,ds=\frac{1}{2}|y'(t)|^2-\frac{1}{2}|y_k^1|^2. \end{aligned}$$

From $H(A)$ and Remark 9 we obtain

9 $$\begin{aligned} \frac{1}{c_2}\int \limits _0^t\Vert y'(s)\Vert _V^p\,ds-t|\Omega |\le \int \limits _0^t\langle A_k(s,y'(s)),y'(s) \rangle \,ds. \end{aligned}$$

Since $B_k$ is linear, symmetric and monotone, it follows that

$$\begin{aligned} \int \limits _0^t\langle B_k y(s),y'(s) \rangle \,ds=\frac{1}{2}\int \limits _0^t\frac{d}{ds}\langle B_k y(s),y(s) \rangle \,ds \end{aligned}$$

10 $$\begin{aligned} =\frac{1}{2}\langle B_ky(t),y(t) \rangle -\frac{1}{2}\langle B_ky_k^0,y_k^0 \rangle \ge -\frac{1}{2}M\Vert y_k^0\Vert ^2. \end{aligned}$$

From ${H(J^1)} \mathrm{(ii)}$ we obtain

11 $$\begin{aligned} \int \limits _0^t\langle \xi (s),\iota y'(s) \rangle _{{Z}^*\times Z}\,ds\ge c_4t-c_5\Vert \iota \Vert ^p\int \limits _0^t\Vert y'(s)\Vert ^p\,ds. \end{aligned}$$

In order to estimate the last term of left hand side of (7), we will use the relation

12 $$\begin{aligned} y(s)=y_k^0+\int \limits _0^sy'(\tau )\,d\tau \,\,\,\mathrm{{for \ all}}\,\,\, s\in (0,T) \end{aligned}$$

and the fact that for all $a,b>0$, $p>1$ there exists a function $\tilde{c}:\mathbb {R}_+\rightarrow \mathbb {R}_+$ such that for all $\varepsilon >0$

13 $$\begin{aligned} (a+b)^p\le (1+\varepsilon )a^p+\tilde{c}(\varepsilon )b^p. \end{aligned}$$

From ${H(J^2)} \mathrm{(i)}$ and (12) we obtain

14 $$\begin{aligned}&\displaystyle \left| \int \limits _0^t\langle \zeta (s),\iota y'(s) \rangle _{Z}\,ds\right| \le \int \limits _0^t\Vert \zeta (s)\Vert _{Z^*}\Vert \iota y'(s)\Vert _Z\,ds\nonumber \\&\displaystyle \le \int \limits _0^t c_6\left( 1+\Vert \iota y(s)\Vert _Z^{p-1}\right) \Vert \iota y'(s)\Vert _Z\,ds\nonumber \\&\displaystyle \le \int \limits _0^t c_6\left( 1+\left\| \iota y_k^0+\int \limits _0^s\iota y'(\tau )\,d\tau \right\| _Z^{p-1}\right) \Vert \iota y'(s)\Vert _Z\,ds\nonumber \\&\displaystyle \le \int \limits _0^t c_6\left( 1+\left( \Vert \iota y_k^0\Vert _Z+\int \limits _0^s\Vert \iota y'(\tau )\Vert _Z\,d\tau \right) ^{p-1}\right) \Vert \iota y'(s)\Vert _Z\,ds. \end{aligned}$$

Using (13) and the Jensen inequality, we obtain

$$\begin{aligned}&\displaystyle \left( \Vert \iota y_k^0\Vert _Z+\int \limits _0^s\Vert \iota y'(\tau )\Vert _Z\,d\tau \right) ^{p-1}\!\le \! (1\!+\!\varepsilon )\left( \int \limits _0^s\Vert \iota y'(\tau )\Vert _Z\,d\tau \right) ^{p-1}\!+\!\tilde{c}(\varepsilon )\Vert \iota y_k^0 \Vert _Z^{p-1}\\&\displaystyle \le (1+\varepsilon )t^{p-2}\int \limits _0^t\Vert \iota y'(s)\Vert _Z^{p-1}\,ds+\tilde{c}(\varepsilon )\Vert \iota y_k^0 \Vert _Z^{p-1}. \end{aligned}$$

Combining the last inequality with (14), we obtain

$$\begin{aligned} \left| \int \limits _0^t\langle \zeta (s),\iota y'(s) \rangle _{Z}\,ds\right| \le c_6(1+\tilde{c}(\varepsilon )\Vert \iota y_k^0 \Vert _Z^{p-1})\int \limits _0^t\Vert \iota y'(s)\Vert _Z\,ds \end{aligned}$$

15 $$\begin{aligned} +c_6(1+\varepsilon )t^{p-2}\int \limits _0^t\Vert \iota y'(s)\Vert _Z^{p-1}\,ds\int \limits _0^t\Vert \iota y'(s)\Vert _Z\,ds. \end{aligned}$$

After simple calculations we obtain,

16 $$\begin{aligned} c_6(1+\tilde{c}(\varepsilon )\Vert \iota y_k^0 \Vert _Z^{p-1})\int \limits _0^t\Vert \iota y'(s)\Vert _Z\,ds\le \varepsilon \int \limits _0^t\Vert \iota y'(s)\Vert _Z^p\,ds+\bar{d}(\varepsilon )+\tilde{d}(\varepsilon )\Vert \iota y_k^0\Vert _Z^p, \end{aligned}$$

17 $$\begin{aligned} \int \limits _0^t\Vert \iota y'(s)\Vert _Z^{p-1}\,ds\int \limits _0^t\Vert \iota y'(s)\Vert _Z\,ds\le t\int \limits _0^t\Vert \iota y'(s)\Vert _Z^p\,ds, \end{aligned}$$

where $\bar{d}(\varepsilon ), \tilde{d}(\varepsilon ) > 0$. From (15) to (17) we obtain for any $\varepsilon >0$

18 $$\begin{aligned} \left| \int \limits _0^t\langle \zeta (s),\iota y'(s) \rangle _{Z}\,ds\right| \le \left( \Vert \iota \Vert ^p c_6t^{p-1}+\varepsilon \right) \int \limits _0^t\Vert y'(s)\Vert ^p\,ds+\hat{c}(\varepsilon )+\tilde{d}(\varepsilon )\Vert \iota y_k^0\Vert _Z^p \end{aligned}$$

with $\hat{c}(\varepsilon )>0$. In order to estimate the right hand side of (7), we use the Young inequality with $\varepsilon >0$ and obtain

19 $$\begin{aligned}&\int \limits _0^t\langle f_k(s),y'(s) \rangle \,ds+\int \limits _0^t\langle (C_ku)(s),y'(s) \rangle \,ds\nonumber \\&\le \varepsilon \int \limits _0^t\Vert y'(s)\Vert ^p\,ds+c(\varepsilon )\left( \Vert f_k\Vert ^q_{\mathcal {V}^*}+\Vert C_k\Vert ^q_{\mathcal {L}(\mathcal {U},\mathcal {V}^*)}\Vert u\Vert ^q_\mathcal {U}\right) . \end{aligned}$$

Combining (8), (10), (11), (18) and (19), we obtain

20 $$\begin{aligned}&\frac{1}{2}|y'(t)|^2+\left( \frac{1}{c_2}-c_5\Vert \iota \Vert ^p-c_6\Vert \iota \Vert ^pt^{p-1}-2\varepsilon \right) \int \limits _0^t\Vert y'(s)\Vert ^p\,ds \\&\quad \le t|\Omega |+\frac{1}{2}M\Vert y_k^0\Vert ^2-c_4t+\hat{c}(\varepsilon )+\bar{d}(\varepsilon )\Vert \iota \Vert ^p\Vert y^0_k\Vert ^p\nonumber \\&\qquad +\frac{1}{2}|y_k^1|^2+c(\varepsilon )\Vert f_k\Vert _\mathcal{V^* }^q+c(\varepsilon )\Vert C_k\Vert ^q_{\mathcal{L}(\mathcal{U}; \mathcal{V}^*) }\Vert u\Vert ^q_\mathcal{U}.\nonumber \end{aligned}$$

Hence due to $(H_1)$, we can choose $\varepsilon >0$ such that the coefficient in front of $\int _0^t\Vert y'(s)\Vert ^p\,ds$ is positive, getting

21 $$\begin{aligned} \Vert y'\Vert ^2_{C(0,T;H)}+\Vert y'\Vert ^p_\mathcal{V}\le C(1+\Vert y^0_k\Vert ^p+|y^1_k|^2+\Vert C_k\Vert ^q_{\mathcal{L}(\mathcal{U}; \mathcal{V}^*) }\Vert u\Vert ^q_\mathcal{U}+\Vert f_k\Vert _\mathcal{V^* }^q), \end{aligned}$$

where the constant $C$ depends on the problem data and $T$ but it is independent on the initial conditions and $k$. From the formula

$$\begin{aligned} y(t)=y^0_k+\int \limits _0^ty'(s)ds\,\,\,\mathrm{{for \ all}}\,\,\,t\in [0,T] \end{aligned}$$

by a direct calculation we obtain

$$\begin{aligned} \Vert y(t)\Vert ^p\le C(\Vert y_k^0\Vert ^p+\Vert y'\Vert ^p_\mathcal{V})\,\,\,\mathrm{{for \ all}}\,\,\, t\in [0,T] , \end{aligned}$$

with the constant $C>0$. Thus, using (21), we have

22 $$\begin{aligned} \Vert y\Vert _{C(0,T;V)}^p\le C(1+|y^1_k|^2+\Vert C_k\Vert ^q_{\mathcal{L}(\mathcal{U}; \mathcal{V}^*) }\Vert u\Vert ^q_\mathcal{U}+\Vert y^0_k\Vert ^p+\Vert f_k\Vert _\mathcal{V^* }^q), \end{aligned}$$

with $C>0$. Moreover, since $y$ solves $(P)_k$, from $H(A)$, Remark 9, $H(B)$, $H(J^1)\mathrm{(i)}$, $H(J^2)\mathrm{(i)}$, $H(C)$ and $(H_0)$ we obtain

23 $$\begin{aligned} \Vert y''\Vert ^q_\mathcal{V^*}\le C(1+\Vert y\Vert ^p_{C(0,T,V)}+\Vert y'\Vert ^p_\mathcal{V}+\Vert C_k\Vert ^q_{\mathcal{L}(\mathcal{U}; \mathcal{V}^*) }\Vert u\Vert ^q_\mathcal{U}+\Vert f_k\Vert _\mathcal{V^* }^q). \end{aligned}$$

The assertion follows from (21) to (23). $\square$

We introduce the family of mappings ${\mathcal {K}}_k:{\mathcal {V}}\rightarrow C(0,T;V)$ by means of the formula

$$\begin{aligned} ({\mathcal {K}}_k y)(t)=y_k^0+\int \limits _0^t y(s) ds\,\,\,\text {for}\,\,\,y\in \mathcal{V}\,\,\,\text {and}\,\,\,t\in [0,T]. \end{aligned}$$

Using this definition, the problem $(P)_k$ can be equivalently rewritten as follows

$$\begin{aligned} {\left\{ \begin{array}{ll} z'(t) + (\mathcal{A}_k z)(t) + (\mathcal{B}_k {\mathcal {K}}_k z)(t) + \iota ^* \partial J^1_k (\iota z(t)) \\ + \iota ^* \partial J^2_k (\iota (\mathcal {K}_k z) (t)) \ni f_k(t) + (C_k u)(t) \ \ \text {a.e.}\, t\in (0,T)\\ z(0)=y_k^1,\ \ z\in \mathcal{W}_{pq}, \end{array}\right. }\quad \quad (P)_k \end{aligned}$$

for $k \in {\bar{\mathbb {N}}}$, where $\mathcal{A}_k :\mathcal{V} \rightarrow \mathcal{V^*}$ and $\mathcal{B}_k :\mathcal{V} \rightarrow \mathcal{V^*}$ are the Nemitsky operators corresponding to $A_k$, and $B_k$, respectively, i.e., $(\mathcal{A}_k v)(t)=A_k(t,v(t))$, $(\mathcal{B}_k v)(t)=B_k(v(t))$ for $v\in \mathcal {V}$ and $t\in [0,T]$.

Now we formulate the existence theorem for the problem $(P)_k$, $k\in \mathbb {N}$. Its proof is analogous to the existence proof of [38], and therefore it will be sketched only briefly here.

#### **Theorem 14**

If the assumptions ${H(A)}$, ${H(B)}\mathrm{(i)}$, ${H(J^1)}$, ${H(J^2)}$, ${(H_0)}$, ${H(C)}$ and ${(H_1)}$ hold, then the problem $(P)_k$, $k\in \bar{\mathbb {N}}$ admits a solution.

#### *Proof*

Let us fix $k\in \bar{\mathbb {N}}$. We will proceed in two steps.

**Step 1.** First we assume that $y_k^1\in V$ and introduce the operators ${\mathcal {A}}_k^1, {\mathcal {B}}_k^1: {\mathcal {V}}\rightarrow \mathcal {V^*}$, $\mathcal {N}_k, \mathcal {M}_k:{\mathcal {V}}\rightarrow 2^{{\mathcal {V^*}}}$ given by

24 $$\begin{aligned} {\mathcal {A}}_k^1v={\mathcal {A}}_k(v+y^1_k)\,\,\,\text {for}\,\,\,v\in {\mathcal {V}}, \end{aligned}$$

25 $$\begin{aligned} {\mathcal {B}}_k^1v={\mathcal {B}}({\mathcal {K}}_k(v+y_k^1))\,\,\,\text {for}\,\,\,v\in {\mathcal {V}}, \end{aligned}$$

26 $$\begin{aligned} {\mathcal {N}}_k v=\{w\in {\mathcal {V}}^*:\,\,w(t)\in \iota ^* \partial J^1_k (\iota (v(t)+y^1_k))\,\,\text {a.e.}\, t\in (0,T)\}\,\,\,\text {for}\,\,\,v\in \mathcal{V}, \end{aligned}$$

27 $$\begin{aligned} {\mathcal {M}}_k v=\{w\in {\mathcal {V}}^*:\,\,w(t)\in \iota ^* \partial J^2_k (\iota {\mathcal {K}}_k(v+y^1_k)(t))\,\,\text {a.e.}\, t\in (0,T)\}\,\,\,\text {for}\,\,\,v\in \mathcal{V}.\nonumber \\ \end{aligned}$$

We also consider the operator $L: D(L)\subset {\mathcal {V}}\rightarrow {\mathcal {V}}^*$ defined by $Lv=v'$ with $D(L)=\{v\in {\mathcal {W}}:v(0)=0\}$ and observe that $z\in {\mathcal {W}}_{pq}$ solves the problem $(P)_k$ if and only if $z-y_k^1\in D(L)$ solves the following one:

28 $$\begin{aligned} Lz+{\mathcal {T}}_kz\ni f_k+Cu_k, \end{aligned}$$

where the operator ${\mathcal {T}}_k:{\mathcal {V}}\rightarrow 2^{{\mathcal {V}}^*}$ is given by

$$\begin{aligned} {\mathcal {T}}_kz={\mathcal {A}}^1_kz+{\mathcal {B}}^1_kz+{\mathcal {N}}_k z+{\mathcal {M}}_k z\,\,\,\text {for}\,\,\,z\in \mathcal{V}. \end{aligned}$$

Recall (see e.g. [51], Proposition 32.10, p. 855) that $L$ is linear, densely defined and maximal monotone operator. Moreover, we will prove that for each $k\in \mathbb {N}$, the operator ${\mathcal {T}}_k$ is bounded, coercive and $L$-generalized pseudomonotone. The solvability of the Problem (28) follows then from Proposition 7. We will state the following four lemmas on the properties of the operators ${\mathcal {A}}^1_k, {\mathcal {B}}^1_k, {\mathcal {N}}_k$ and ${\mathcal {M}}_k$. The proofs of these lemmas are analogous to the proofs of the lemmas $7$, $8$, $9$ and $12$ of [38] (see also Remark 11). $\square$

#### **Lemma 15**

If $H(A)$ holds and $y_k^1\in V$, then for each $k\in \bar{\mathbb {N}}$ the operator ${\mathcal {A}}_k^1$ defined by (24) satisfies:

- $\Vert {\mathcal {A}}_k^1v\Vert _{{\mathcal {V}}^*}^q\le d_1\Vert v\Vert _{{\mathcal {V}}}^p+d_2$ for all $v\in {\mathcal {V}}$ with $d_1,d_2>0$;
- $\langle \langle {\mathcal {A}}_k^1v,v \rangle \rangle _{{{\mathcal {V}}}^*\times {\mathcal {V}}}\ge \left( \frac{1}{c_2}-\varepsilon \right) \Vert v\Vert _{{\mathcal {V}}}^p-d_3(\varepsilon )$ for all $v\in {\mathcal {V}}$, $\varepsilon >0$, where $d_3(\varepsilon )>0$;
- ${\mathcal {A}}_k^1$ is monotone and hemicontinuous (so also demicontinuous);
- ${\mathcal {A}}_k^1$ is $L$-generalized pseudomonotone;
- For every $\{v_n\}\subset \mathcal{W}_{pq}$ with $v_n\rightarrow v$ weakly in $\mathcal{W}_{pq}$ and $\limsup _{n\rightarrow \infty }\langle \langle {\mathcal {A}}_kv_n,v_n-v \rangle \rangle _{\mathcal{V}^*\times \mathcal V}\le 0$, it follows that ${\mathcal {A}}_k v_n\rightarrow {\mathcal {A}}_k v$ weakly in $\mathcal{V}^*$.

#### **Lemma 16**

If $H(B)\mathrm{(i)}$ holds and $y_k^1\in V$, then for each $k\in \bar{\mathbb {N}}$ the operator ${\mathcal {B}}_k^1$ defined by (25) satisfies:

- $\Vert {\mathcal {B}}_k^1v\Vert _{{\mathcal {V}}}\le d_{4}(1+\Vert v\Vert _{{\mathcal {V}}})$ for all $v\in {\mathcal {V}}$ with $d_4>0$;
- $\Vert {\mathcal {B}}_k^1v-{\mathcal {B}}_k^1w\Vert _{{\mathcal {V}}^*}\le d_{5}\Vert v-w\Vert _{{\mathcal {V}}}$ for all $v,w\in {\mathcal {V}}$ with $d_{5}>0$;
- $\langle \langle {\mathcal {B}}_k^1v,v \rangle \rangle _{{{\mathcal {V}}}^*\times {\mathcal {V}}}\ge -d_{6}\Vert v\Vert _{{\mathcal {V}}}-d_{7}$ for all $v\in {\mathcal {V}}$ with $d_{6}\ge 0$ and $d_{7}\ge 0$;
- ${\mathcal {B}}_k^1$ is monotone and weakly continuous.

Moreover, if $H(B)\mathrm{(i)}$ holds and $y_k^1\in H$, then the Nemitsky operator ${\mathcal {B}}_k$ corresponding to $B_k$ satisfies

(f) $\langle \langle {\mathcal {B}}_kv-{\mathcal {B}}_kw,v'-w' \rangle \rangle _{{{\mathcal {V}}}^*\times {\mathcal {V}}}\ge 0$ for all $v, w\in {\mathcal {W}}$ such that $v(0)=w(0)$;
(g) ${\mathcal {B}}_k$ is weakly continuous as a mapping from $\mathcal{W}_{pq}$ to $\mathcal{V}^*$.

#### **Lemma 17**

If $H(J^1)$ holds and $y_k^1\in V$, then for each $k\in \bar{\mathbb {N}}$ the operator ${\mathcal {N}}_k$ defined by (26) satisfies:

- for each $v\in {\mathcal {V}}$, ${\mathcal {N}}_kv$ is a nonempty, convex and weakly compact subset of ${\mathcal {V}}^*$;
- $\Vert w\Vert _{{\mathcal {V}}^*}^q\le d_{8}(1+\Vert v\Vert _{{\mathcal {V}}}^p)$ for all $w\in {\mathcal {N}}_kv$ and $v\in {\mathcal {V}}$, with $d_{8}>0$;
- $\langle \langle w,v \rangle \rangle _{{{\mathcal {V}}}^*\times {\mathcal {V}}}\ge -d_9(\varepsilon )-(c_5\Vert \iota \Vert ^p+\varepsilon )\Vert v\Vert _{{\mathcal {V}}}^p$ for all $w\in {\mathcal {N}}_kv$ and $v\in {\mathcal {V}}$ and $\varepsilon >0$ with $d_9(\varepsilon )>0$;
- if $v_n\rightarrow v$ strongly in $\mathcal {Z}$, $w_n\rightarrow w$ weakly in $\mathcal {Z}^*$ and $w_n\in {\mathcal {N}}_k v_n$, then $w\in {\mathcal {N}}_k v$.

#### **Lemma 18**

If $H(J^2)$ holds and $y_k^1\in V$, then for each $k\in \bar{\mathbb {N}}$ the operator ${\mathcal {M}}_k$ defined by (27) satisfies:

- for each $v\in {\mathcal {V}}$, ${\mathcal {M}}_kv$ is a nonempty convex and weakly compact subset of ${\mathcal {V}}^*$;
- $\Vert w\Vert _{{\mathcal {V}}^*}^q \le d_{10}(1+\Vert v\Vert _{{\mathcal {V}}}^p)$ for all $w\in {\mathcal {M}}_k v$ and $v\in {\mathcal {V}}$, with $d_{10}>0$;
- $\langle \langle w,v \rangle \rangle _{{{\mathcal {V}}}^*\times {\mathcal {V}}}\ge -\left( c_6\Vert \iota \Vert ^p T^{p-1}+\varepsilon \right) \Vert v\Vert _{{\mathcal {V}}}^p-d_{11}(\varepsilon )$ for all $w\in {\mathcal {M}}_k$ and $v\in {\mathcal {V}}$;
- if $v_n\rightarrow v$ strongly in $\mathcal {Z}$, $w_n\rightarrow w$ weakly in $\mathcal {Z}^*$ and $w_n\in {\mathcal {M}}_k v_n$ then $w\in {\mathcal {N}}_k v$.

We continue the proof of Theorem 14.

#### **Claim 1**

$\mathcal {T}_k$ is bounded. This follows directly from Lemmata 15 (a), 16 (a), (b) and (b).

#### **Claim 2**

$\mathcal {T}_k$ is coercive. This follows directly from Lematta 15 (b), 16 (c), (c), (c) and from ${(H_1)}$.

#### **Claim 3**

$\mathcal {T}_k$ is $L-$generalized pseudomonotone. It can be proved by the argument that exactly follows the lines of the proof of Theorem 6 in [38]. We omit the proof for brevity.

**Step 2.** Now we pass to the more general case and assume that $y^1_k\in H$. The proof is analogous to Step 2 in the proof of Theorem 6 in [38]. However, we provide the proof, since we deal with more general case involving a sum of two subdifferentials. Since $V\subset H$ is dense, we can find a sequence $\{y^{1n}_k\}\subset V$ such that $y^{1n}_k\rightarrow y^1_k$ in $H$, as $n\rightarrow \infty$ (index $k$ is now fixed). Consider a solution $y_n$ of the problem $(P)_k$ when $y^1_k$ is replaced by $y^{1n}_k$, i.e., a solution of the following problem

$$\begin{aligned} {\left\{ \begin{array}{ll} y_n''(t) + (\mathcal{A}_k y_n')(t) + (\mathcal{B}_k y_n)(t) +\\ \iota ^* \partial J^1_k (\iota y_n'(t)) + \iota ^* \partial J^2_k (\iota y_n(t)) \ni f_k(t) + (C_k u)(t) \ \ \text {a.e.}\, t\in (0,T)\\ y_n(0) = y_k^0,\ \ y_n'(0)=y_k^{1n},\ \ y_n\in \mathcal{Y}. \end{array}\right. }\quad \quad (P)_k^n \end{aligned}$$

The existence of $y_n$, for $n\in \mathbb {N}$ follows from the first part of the proof. We have

29 $$\begin{aligned} y_n''(t)\!+\!A_k(t,y_n'(t))\!+\!B_ky_n(t)\!+\!\iota ^*\xi _n(t)\!+\!\iota ^*\zeta _n(t)\!=\!f_k(t)\!+\!(C_ku)(t)\,\,\,\mathrm{for \ a.e.}\,\, t\!\in \! (0,T),\nonumber \\ \end{aligned}$$

or equivalently

$$\begin{aligned} y_n''+\mathcal {A}_k y_n'+\mathcal {B}_k y_n+\iota ^*\xi _n+\iota ^*\zeta _n=f_k+C_ku\,\,\,\text {in}\,\,\, \mathcal {V}^*. \end{aligned}$$

where

30 $$\begin{aligned} \xi _n(t)\in \partial J^1_k (\iota y_n'(t))\,\,\,\,\mathrm{{for \ a.e.}}\,\, t\in (0,T) \end{aligned}$$

and

31 $$\begin{aligned} \zeta _n(t)\in \partial J^2_k (\iota y_n(t))\,\,\,\,\mathrm{{for \ a.e.}}\,\, t\in (0,T). \end{aligned}$$

From Lemma 13, since all terms in the right-hand side of (6) excluding $y^1_k$ do not depend on $n$, we have

$$\begin{aligned} \Vert y_n\Vert _{C(0,T;V)}^p + \Vert y'_n\Vert ^2_{C(0,T;H)}+\Vert y'_n\Vert ^p_\mathcal{V} + \Vert y''_n\Vert ^q_\mathcal{V^*} \le C(1+|y^{1n}_k|^2). \end{aligned}$$

Since $\{y^{1n}_k\}$ is bounded in $H$ also $\{y_n\}$ and $\{y_n'\}$ are bounded in $\mathcal{V}$ and $\mathcal{W}_{pq}$, respectively. So passing to a subsequence, we have

32 $$\begin{aligned} y_n{\buildrel {\Xi } \over \longrightarrow }y. \end{aligned}$$

We will show that $y$ is a solution of the problem $(P)_k$. From (32) we also have $y_n\rightarrow y$ weakly in $\mathcal{W}_{pq}$. From the continuity of embedding $\mathcal{W}_{pq}\subset C(0,T;H)$ it follows that $y_n(0)\rightarrow y(0)$ weakly in $H$ and since $y_n(0)=y^0_k$ for all $n\in \mathbb {N}$ we conclude that $y(0)=y^0_k$. Moreover, $y_n'(0)\rightarrow y'(0)$ weakly in $H$ and since $y_n'(0)=y^{1n}_k\rightarrow y^1_k$ strongly in $H$, it follows that $y'(0)=y^1_k$. From Lemma 16 and (32), it follows that

33 $$\begin{aligned} \mathcal {B}_k y_n\rightarrow \mathcal {B}_k y\,\,\, \mathrm{{weakly\ in}}\,\,\, \mathcal{V}^*. \end{aligned}$$

Now we pass to the limit in (30) and (31). Since $y_n\rightarrow y$ and $y_n'\rightarrow y'$ weakly in $\mathcal{W}_{pq}$ and $\iota :\mathcal{W}_{pq}\rightarrow \mathcal{Z}$ is compact it follows that $\iota y_n\rightarrow \iota y$ and $\iota y'_n\rightarrow \iota y'$ strongly in $\mathcal{Z}$. From growth conditions $H(J^1)\mathrm{(i)}$ and $H(J^2)\mathrm{(i)}$, it follows that $\{\xi _n\}$ and $\{\zeta _n\}$ are bounded in $\mathcal{Z}^*$. From the reflexivity of this space, we have for a subsequence $\xi _n\rightarrow \xi$ and $\zeta _n\rightarrow \zeta$ weakly in $\mathcal{Z}^*$. From the convergence theorem of Aubin and Cellina (see [4]), we have

$$\begin{aligned} \xi (t)\in \partial J^1_k (\iota y'(t))\,\,\,\text {and}\,\,\,\zeta (t)\in \partial J^2_k (\iota y(t)) \,\,\,\mathrm{{for \ a.e.}}\,\, t\in (0,T). \end{aligned}$$

Since $\iota ^*:\mathcal{Z}^*\rightarrow \mathcal{V}^*$ is linear and continuous, and hence weakly continuous, we have

34 $$\begin{aligned} \iota ^*\xi _n\rightarrow \iota ^*\xi \,\,\,\text {and}\,\,\,\iota ^*\zeta _n\rightarrow \iota ^*\zeta \,\,\,\mathrm{{weakly\ in}} \,\,\,\mathcal{V}^*. \end{aligned}$$

In order to pass to the limit in the term $\mathcal {A}_k y_n'$, we will show that

35 $$\begin{aligned} \limsup _{n\rightarrow \infty }\langle \langle {\mathcal {A}}_ky'_n,y'_n-y' \rangle \rangle _{\mathcal{V}^*\times \mathcal V}\le 0 \end{aligned}$$

and use Lemma 15 $(e)$. Since $\lim _{n\rightarrow \infty }\langle \langle f_k+C_ku,y_n'-y' \rangle \rangle _{\mathcal{V}^*\times \mathcal V}=0$ and

$\lim _{n\rightarrow \infty }\langle \langle \xi _n+\zeta _n,\iota y_n'-\iota y' \rangle \rangle _{\mathcal{Z}^*\times \mathcal Z}=0$ from (29), we have

$$\begin{aligned} \limsup _{n\rightarrow \infty }\langle \langle {\mathcal {A}}_ky'_n,y'_n-y' \rangle \rangle _{\mathcal{V}^*\times \mathcal V} \end{aligned}$$

36 $$\begin{aligned} \le \limsup _{n\rightarrow \infty }\langle \langle y''_n,y'-y'_n \rangle \rangle _{\mathcal{V}^*\times \mathcal V}+\limsup _{n\rightarrow \infty }\langle \langle {\mathcal {B}}_ky_n,y'-y'_n \rangle \rangle _{\mathcal{V}^*\times \mathcal V}. \end{aligned}$$

We calculate

$$\begin{aligned} \limsup _{n\rightarrow \infty }\langle \langle y''_n,y'-y'_n \rangle \rangle _{\mathcal{V}^*\times \mathcal V}=\limsup _{n\rightarrow \infty }\langle \langle y''_n-y'',y'-y'_n \rangle \rangle _{\mathcal{V}^*\times \mathcal V} \end{aligned}$$

37 $$\begin{aligned} =\frac{1}{2}\limsup _{n\rightarrow \infty }(|y_n'(0)-y'(0)|^2-|y_n'(T)-y'(T)|^2)\le \frac{1}{2}\lim _{n\rightarrow \infty }|y^{1n}_k-y^1_k|^2 = 0. \end{aligned}$$

On the other hand, by the Lemma 16 (f), we have

$$\begin{aligned} \limsup _{n\rightarrow \infty }\langle \langle \mathcal {B}_k y_n,y'-y'_n \rangle \rangle _{\mathcal{V}^*\times \mathcal V}=\lim _{n\rightarrow \infty }\langle \langle \mathcal {B}_k y,y'-y'_n \rangle \rangle _{\mathcal{V}^*\times \mathcal V} \end{aligned}$$

38 $$\begin{aligned} +\limsup _{n\rightarrow \infty }\langle \langle \mathcal {B}_k y_n-\mathcal {B}_k y,y'-y'_n \rangle \rangle _{\mathcal{V}^*\times \mathcal V}\le 0. \end{aligned}$$

From (36) to (38), we obtain (35), so we conclude that

39 $$\begin{aligned} {\mathcal {A}}_ky'_n\rightarrow {\mathcal {A}}_ky'\,\,\,\mathrm{{weakly \ in}}\,\,\,\mathcal{V}^*. \end{aligned}$$

From (32), (33),(34) and (39) it follows that $y$ solves $(P)_k$. The proof is complete.

#### **Theorem 19**

If, in addition to the assumptions of the Theorem 14, we admit $p=2$ and for $k\in \bar{\mathbb {N}}$:

40 $$\begin{aligned}&(a_k(t,x,\xi ) - a_k(t,x,\eta ), \xi - \eta )_{{\mathbb {R}}^N} \ge b_1 |\xi - \eta |^2 \,\,\mathrm{{a.e.\ in}}\,\, Q \,\, \mathrm {for \ all}\,\, \xi , \eta \in {\mathbb {R}}^N,\nonumber \\\end{aligned}$$

41 $$\begin{aligned}&\langle \eta _1-\eta _2,z_1-z_2 \rangle _{{Z}^*\times Z}\ge -b_2\Vert z_1-z_2\Vert ^2\,\,\mathrm{{for \ all}}\,\,z_i\in Z, \eta _i\in \partial J_k^1(z_i),\,\,i=1,2,\nonumber \\\end{aligned}$$

42 $$\begin{aligned}&J^2_k\in C^2(Z)\,\,\mathrm{{with}}\,\,\Vert D^2J_k^2(z)\Vert _{{\mathcal {L}}(Z;Z^*)}\le b_3\,\,\mathrm{{for \ all}}\,\, z\in Z,\nonumber \\ \end{aligned}$$

with $b_1,b_2,b_3>0$ and $b_1>b_2\Vert \iota \Vert ^2$, then the solution of the problem $(P)_k$ is unique.

The proof of Theorem 19 is based on a standard technique and follows from a simple direct calculation and application of the Gronwall lemma.

### Sensitivity of Solution Sets for $(P)_k$

In this section we provide the result of the sensitivity of the solution set of the second order subdifferential inclusion.

We assume that $\mathfrak {u}\in \mathfrak {U}\subset {\mathcal {U}}\times V\times H$, where $\mathfrak {U}$ is the set of admissible controls. Moreover, for a sequence of controls $\{\mathfrak {u}_k\}\subset \mathfrak {U}$, $k\in \overline{\mathbb {N}}$ we say that $(u_k,y^0_k,y^1_k)=\mathfrak {u}_k\mathop {\longrightarrow }\limits ^{s-\mathfrak {U}}\mathfrak {u_\infty }=(u_\infty ,y^0_\infty ,y^1_\infty )$ if $u_k\mathop {\longrightarrow }\limits ^{s-{\mathcal {U}}} u_\infty$, $y^0_k\mathop {\longrightarrow }\limits ^{s-V} y^0_\infty$, and $y^1_k\mathop {\longrightarrow }\limits ^{s-H} y^1_\infty$, as $k\rightarrow \infty$. Let us define the multivalued mappings $\mathcal{S}_k:\mathfrak {U}\rightarrow 2^\mathcal{Y}$, which assign to the control $\mathfrak {u}\in \mathfrak {U}$ the set of all solutions of the problems $(P)_k$ corresponding to this control, where $k\in \bar{\mathbb {N}}$. First we observe that under the hypotheses of Theorem 14, the mappings ${\mathcal {S}}_k$ have nonempty values. We prove the following theorem.

#### **Theorem 20**

Under the hypotheses $H(A)$, $H(B)$, $H(J^1)$, $H(J^2)$, $H(C)$, $(H_0)$ and $(H_1)$, from any sequence $\{y_k\}$, $k\in \mathbb {N}$ such that $y_k\in \mathcal{S}_k(\mathfrak {u}_k)$ where $\mathfrak {u}_k{\buildrel s-\mathfrak {U} \over \longrightarrow } \mathfrak {u}_\infty$, one can extract a convergent subsequence $y_{k_n} {\buildrel {\Xi } \over \longrightarrow }y_\infty$, where $y_\infty \in \mathcal{S}_{\infty }(\mathfrak {u}_\infty )$, that is

43 $$\begin{aligned} \displaystyle K(\Xi -{\mathcal {Y}})-\limsup \mathcal{S}_k(\mathfrak {u}_k) \subset \mathcal{S}_\infty (\mathfrak {u}_\infty ) \ \mathrm{{for\ every}} \ \mathfrak {u}_k \ {\buildrel {s-\mathfrak {U}} \over \longrightarrow } \ \mathfrak {u}_\infty . \end{aligned}$$

Moreover, if the limit problem $(P)_\infty$ has a unique solution (for example, if the assumptions of Theorem 19 hold for $k=\infty$), then we also have

44 $$\begin{aligned} \displaystyle \mathcal{S}_\infty (\mathfrak {u}_\infty ) \subset K(\Xi -{\mathcal {Y}})-\liminf \mathcal{S}_k(\mathfrak {u}_k), \end{aligned}$$

so in this case $\displaystyle \mathcal{S}_k(\mathfrak {u}_k) \ \ {\buildrel K(\Xi -{\mathcal {Y}}) \over \longrightarrow } \ \ \mathcal{S}_\infty (\mathfrak {u}_\infty )$, as $k\rightarrow \infty$.

Before we give the proof of the Theorem 20, we prove a lemma, which to the best of our knowledge is a new result.

#### **Lemma 21**

Let $Z$ be a separable and reflexive Banach space, and let $\displaystyle J_k :Z \rightarrow {\mathbb {R}}$ $k\in \bar{\mathbb {N}}$ be a family of locally Lipschitz functions such that $||\partial J_k(z)||_{Z^*} \le c(1 + ||z||_Z^{p-1})$ for all $z \in Z$ with some $c > 0$ independent of $k$ and

45 $$\begin{aligned} K(s-Z, w-Z^*) -\limsup _{k\rightarrow \infty } Gr \, \partial J_k \subset Gr \, \partial J_\infty . \end{aligned}$$

Then

- for every sequence $z_k\rightarrow z$ strongly in $Z$ and for every $v\in Z$ we have
  46 $$\begin{aligned} \limsup _{k\rightarrow \infty } J_k^0(z_k;v)\le J^0_{\infty }(z;v), \end{aligned}$$
- for all sequences $z_k\rightarrow z$ strongly in $\mathcal {Z}$ and $\xi _k\rightarrow \xi$ weakly in $\mathcal {Z}^*$ such that $\xi _k(t)\in \partial J_k(z_k(t))$ for a.e. $t\in (0,T)$ we have $\xi (t)\in \partial J_{\infty }(z(t))$ for a.e. $t\in (0,T)$.

#### *Proof*

For the proof of a) assume that for a subsequence $J^0_k(z_k;v)\rightarrow \alpha \in \mathbb {R}$ (note that due to the growth condition the case $\alpha = \infty$ is excluded here). It is enough to show, that $\alpha \le J^0_{\infty }(z;v)$. From the basic properties of the Clarke subdifferential, we have

$$\begin{aligned} J^0_k(z_k;v)=\max \{\langle \xi ,v \rangle \,\,:\ \xi \in \partial J_k(z_k)\}=\langle \xi _k,v \rangle \ \mathrm{{for}}\ k\in \mathbb {N} \end{aligned}$$

where $\xi _k\in \partial J_k(z_k)$. From the growth condition, since $\{z_k\}$ is bounded, it follows that for a subsequence, we have $\xi _k\rightarrow \xi$ weakly in $Z^*$ for some $\xi \in Z^*$. Hence $\langle \xi _k,v \rangle \rightarrow \langle \xi ,v \rangle =\alpha$. From (45) we have $\xi \in \partial J_{\infty }(z)$, so $\alpha =\langle \xi ,v \rangle \le J^0_{\infty }(z;v)$ and the proof of part a) is complete.

Now we pass to the proof of b). Fix $v\in {\mathcal {Z}}$. Since $z_k\rightarrow z$ strongly in $\mathcal {Z}$, then using Proposition 2.2.41 in [23] for a subsequence $z_{k_n}$, we have $z_{k_n}(t)\rightarrow z(t)$ for a.e. $t\in (0,T)$ with $n\rightarrow \infty$, and for all $n\ge 1$, $\Vert z_{k_n}(t)\Vert _Z\le h(t)$ for a.e. $t\in (0,T)$ with some $h\in L^p(0,T)$. From the growth condition, we have

$$\begin{aligned}&\langle \xi _{k_n}(t),v(t) \rangle _{Z^*\times Z }\le \Vert \xi _{k_n}(t)\Vert _{Z^*}\Vert v(t)\Vert _Z\\&\le c(1+\Vert z_{k_n}(t)\Vert _Z^{p-1})\Vert v(t)\Vert _Z\le c(1+h(t)^{p-1})\Vert v(t)\Vert _Z. \end{aligned}$$

Since the last function belongs to $L^1(0,T)$, we can use the Fatou lemma and obtain

47 $$\begin{aligned} \limsup _{k\rightarrow \infty }\int \limits _0^T\langle \xi _{k_n}(t),v(t) \rangle \,dt\le \int \limits _0^T\limsup _{k\rightarrow \infty }\langle \xi _{k_n}(t),v(t) \rangle \,dt. \end{aligned}$$

Since $\xi _k\rightarrow \xi$ weakly in $\mathcal {Z^*}$, we have

48 $$\begin{aligned} \int \limits _0^T\langle \xi (t),v(t) \rangle dt=\lim _{n\rightarrow \infty }\int \limits _0^T\langle \xi _{k_n}(t),v(t) \rangle dt. \end{aligned}$$

Applying the assertion a) of the Lemma as well as (47), (48), and the definition of the Clarke subdifferential, we obtain

$$\begin{aligned}&\int \limits _0^T\langle \xi (t),v(t) \rangle dt\le \int \limits _0^T\limsup _{n\rightarrow \infty }\langle \xi _{k_n}(t),v(t) \rangle \,dt\\&\quad \le \int \limits _0^T\limsup _{n\rightarrow \infty }J^0_{k_n}(z_{k_n}(t);v(t))\,dt\le \int \limits _0^T J^0_{\infty }(z(t);v(t))\,dt. \end{aligned}$$

We have shown that

49 $$\begin{aligned} \int \limits _0^T \left( J^0_{\infty }(z(t);v(t)) - \langle \xi (t),v(t) \rangle \right) dt\ge 0 \ \ \mathrm{{for \ all}}\ \ v\in {\mathcal {Z}}. \end{aligned}$$

Next, we will show that for all $v\in {\mathcal {Z}}$, the integrand in (49) is nonnegative for a.e. $t\in (0,T)$. Indeed, to the contrary, suppose that for some $v\in {\mathcal {Z}}$, we have $J^0_{\infty }(z(t);v(t)) - \langle \xi (t),v(t) \rangle < 0$ for all $t\in N\subset (0,T)$, where $N$ is of positive measure. Define

$$\begin{aligned} w(t)={\left\{ \begin{array}{ll} v(t)\ &{}\mathrm{{for}}\ t\in N,\\ 0\ &{}\mathrm{{for}}\ t\in (0,T)\setminus N. \end{array}\right. } \end{aligned}$$

Then

$$\begin{aligned} \int \limits _0^T \left( J^0_{\infty }(z(t);w(t)) - \langle \xi (t),w(t) \rangle \right) dt = \int \limits _A \left( J^0_{\infty }(z(t);v(t)) - \langle \xi (t),v(t) \rangle \right) dt < 0, \end{aligned}$$

and, since $w\in {\mathcal {Z}},$ we have a contradiction with (49).

Now, by the separability of $Z$, consider a countable dense subset $\{v_n\}_{n=1}^\infty$ of $Z$. Taking in place of $v$ in (49), the constant functions $w_n \in {\mathcal {Z}}$ defined by $w_n(t) = v_n$ for $t\in (0,T)$, we observe that the inequality $J^0_{\infty }(z(t);v_n) - \langle \xi (t),v_n \rangle \ge 0$ does not hold on the set $N_n\subset (0,T)$ of measure zero. Now, we have $\langle \xi (t),v_n \rangle \le J^0_{\infty }(z(t);v_n)$ for all $n\in \mathbb {N}$ and $t$ belonging to the set of full measure $(0,T) \setminus \bigcup _{n=1}^\infty N_n$. Since $J^0_\infty (z(t);\cdot )$ is locally Lipschitz and hence continuous (see Proposition 2.1.1 in [10]), then, by density, we have $\langle \xi (t),v \rangle \le J^0_{\infty }(z(t);v)$ for all $v\in Z$ on the set of full measure and the assertion follows. $\square$

#### *Proof*

*(of Theorem 20)*. First observe that from Theorem 14, it follows that the sets $\mathcal{S}_k(\mathfrak {u}_k)$ are nonempty for $k\in \bar{\mathbb {N}}.$ Suppose that $y_k\in \mathcal{S}_k(\mathfrak {u}_k)$ for $k\in \mathbb {N}$. From Lemma 13, it follows that $y_k$ is bounded in $C(0,T;V)$, $y_k'$ is bounded in $C(0,T;H)\cap \mathcal{V}$ and $y_k''$ is bounded in $\mathcal{V}^*$. Hence, for a subsequence, we have $y_k {\buildrel \Xi \over \longrightarrow } y_\infty$. It remains to show that $y_\infty \in \mathcal{S}_\infty (\mathfrak {u}_\infty )$. In a standard way, from $y_k {\buildrel \Xi \over \longrightarrow } y_\infty$, it follows that $y_k(0)\rightarrow y_\infty (0)$ weakly in $V$ and $y_k'(0)\rightarrow y_\infty '(0)$ weakly in $H$. Hence, from $(H_0)$, we obtain $y_\infty (0)=y^0_\infty$ and $y_\infty '(0)=y^1_\infty$.

From the fact that $y_k\in \mathcal{S}_k(\mathfrak {u}_k)$, it follows that for a.e. $t\in (0,T)$, we have

50 $$\begin{aligned} y_k''(t) + A_k(t, y_k'(t)) + B_k y_k(t) + \iota ^* \xi _k(t) + \iota ^*\zeta _k(t)= f_k(t) + (C_k u_k)(t), \end{aligned}$$

where $\xi _k(t)\in \partial J^1_k (\iota y_k'(t))$ and $\zeta _k(t)\in \partial J^2_k (\iota y_k(t))$ for a.e. $t\in (0,T)$. From the growth conditions, we know that both $\{\xi _k\}$ and $\{\zeta _k\}$ are bounded in $\mathcal {Z}^*$, so for a subsequences numerated by $k$ again, we have

51 $$\begin{aligned} \xi _k\rightarrow \xi \,\,\,\text {and}\,\,\,\zeta _k\rightarrow \zeta \,\,\,\text {weakly}\,\,\,\text {in}\,\,\,\mathcal{{Z}^*}\,\,\,\text {as}\,\,\,k \rightarrow \infty . \end{aligned}$$

Moreover, since $y_k\rightarrow y_\infty$ and $y_k'\rightarrow y'_\infty$ both weakly in ${\mathcal {W}}_{pq}$, and the Nemytskii operator ${\iota }:{\mathcal {W}}_{pq}\rightarrow {\mathcal {Z}}$ is compact, we have

52 $$\begin{aligned} \iota y_k\rightarrow \iota y_\infty \,\,\,\text {and}\,\,\,\iota y'_k\rightarrow \iota y_\infty ' \,\,\,\text {strongly in}\,\,\,\mathcal{{Z}}\,\,\,\text {as}\,\,\, k \rightarrow \infty . \end{aligned}$$

From (51), (52) and Lemma 21, we obtain

53 $$\begin{aligned} \xi (t)\in \partial J^1_\infty (\iota y_\infty '(t))\,\,\, \text {and}\,\,\, \zeta (t)\in \partial J^2_\infty (\iota y_\infty (t))\,\,\, \text {for a.e.}\ t\in (0,T). \end{aligned}$$

From Remark 9, we may assume, possibly passing to a subsequence, that

54 $$\begin{aligned} a_k(x,t,Dy_k')\rightarrow b(x,t) \,\,\,\text {weakly}\,\,\,\text {in}\,\,\,L^q(Q;\mathbb {R}^N) \end{aligned}$$

with some $b\in L^q(Q;\mathbb {R}^N)$. Let $\eta \in \mathbb {R}^N$ and let $\Omega _0$ be an open set such that $\Omega _0\subset \subset \Omega$, and let $\Delta$ be an open interval with $\Delta \subset \subset (0,T)$. Let $\Phi \in C^\infty _0(\Omega )$, $\Psi \in C^\infty _0((0,T))$ be such that $\Phi |_{\Omega _0}=1$ and $\Psi |_{\Delta }=1$. We define

$$\begin{aligned} v(x,t)=\Phi (x)\Psi (t)(\eta ,x)_{\mathbb {R}^N}\,\,\,\text {for all}\,\,\,(x,t)\in \Omega \times [0,T]. \end{aligned}$$

Let us consider a sequence $\{v_k\}\subset {\mathcal {W}}_{pq}$ of solutions to the following auxiliary problem

55 $$\begin{aligned} v_k'-\text {div}\,a_k(x,t,Dv_k)&= v'(t)-\text {div}\,a_\infty (x,t,Dv)\end{aligned}$$

56 $$\begin{aligned} v_k(0)&= 0. \end{aligned}$$

By $H(A)$, we have

57 $$\begin{aligned} v_k\rightarrow v\,\,\, \text {weakly}\,\,\,\text {in}\,\,\,{\mathcal {W}}_{pq} \end{aligned}$$

58 $$\begin{aligned} a_k(x,t,Dv_k)\rightarrow a_\infty (x,t,\eta )\,\,\, \text {weakly}\,\,\,\text {in}\,\,\, L^q(\Omega _0\times \Delta ;\mathbb {R}^N). \end{aligned}$$

Let $\varphi \in C^\infty _0(\Omega _0\times \Delta )$, $\varphi \ge 0$. The monotonicity of $a_k(x,t,\cdot )$ implies

59 $$\begin{aligned} \int \limits _{\Omega _0\times \Delta }(a_k(x,t,Dy_k')-a_k(x,t,Dv_k),Dy_k'-Dv_k)\varphi \,dxdt\ge 0. \end{aligned}$$

From (50) and (55), we have

$$\begin{aligned}&y_k''-v_k'-\text {div}\,(a_k(x,t,Dy_k')-a_k(x,t,Dv_k))+B_ky_k+\bar{\iota }^*\xi _k+\bar{\iota }^*\zeta _k\\&\quad =f_k+C_ku_k-v'+\text {div}\,a_\infty (x,t,Dv). \end{aligned}$$

Multiplying the last equality by $(y_k'-v_k)\varphi$ and integrating by parts, we obtain

60 $$\begin{aligned}&\int \limits _{\Omega _0\times \Delta }(a_k(x,t,Dy'_k)-a_k(x,t,Dv_k), Dy_k'-Dv_k)\varphi \,dxdt\nonumber \\&\quad =-\int \limits _{\Omega _0\times \Delta }(a_k(x,t,Dy'_k)-a_k(x,t,Dv_k), y_k'-v_k)D\varphi \,dxdt\nonumber \\&\qquad -\langle y_k''-v_k',(y_k'-v_k)\varphi \rangle _{{{\mathcal {V}}}^*\times {\mathcal {V}}}-\langle \xi _k+\zeta _k,\bar{\iota }(y_k'-v_k)\varphi \rangle _{{{\mathcal {Z}}}^*\times {\mathcal {Z}}}\nonumber \\&\qquad +\langle f_k+C_ku_k-v'+\text {div}\,a_\infty (x,t,Dv)-B_ky_k,(y_k'-v_k)\varphi \rangle _{{{\mathcal {V}}}^*\times {\mathcal {V}}}. \end{aligned}$$

We claim that

61 $$\begin{aligned} \langle y_k''-v_k',(y_k'-v_k)\varphi \rangle _{{{\mathcal {V}}}^*\times {\mathcal {V}}}\rightarrow \langle y''-v',(y'-v)\varphi \rangle _{{{\mathcal {V}}}^*\times {\mathcal {V}}}, \,\,\,\text {as}\,\,\,k\rightarrow \infty . \end{aligned}$$

Indeed, let $z_k=y_k'-v_k$ and $z=y'-v$. We know that $z_k\rightarrow z$ weakly in ${\mathcal {W}}_{pq}$ and also strongly in ${\mathcal {H}}$. Since

$$\begin{aligned} \langle z_k',z_k\varphi \rangle _{{{\mathcal {V}}}^*\times {\mathcal {V}}}=-\frac{1}{2}(z_k,z_k\varphi ')_{{\mathcal {H}}}, \end{aligned}$$

we have

$$\begin{aligned} |(z_k,z_k\varphi ')_{{\mathcal {H}}}-(z,z\varphi ')_{{\mathcal {H}}}|\le \Vert z_k\Vert _{{\mathcal {H}}}\Vert (z_k-z)\varphi '\Vert _{{\mathcal {H}}}+\Vert z_k-z\Vert _{{\mathcal {H}}}\Vert z\varphi '\Vert _{{\mathcal {H}}}\rightarrow 0, \end{aligned}$$

which proves (61). Since the embedding ${\mathcal {W}}_{pq}\subset L^p(Q)$ is compact, and moreover, $\iota :{\mathcal {W}}_{pq}\rightarrow {\mathcal {Z}}$ is compact operator, from (57) and $y_k {\buildrel \Xi \over \longrightarrow } y_\infty$, we conclude that

62 $$\begin{aligned}&v_k\rightarrow v\,\,\,\text {and}\,\,\,y'_k\rightarrow y_\infty '\,\,\,\text {strongly}\,\,\text {in}\,\,L^p(Q),\\&\iota v_k\rightarrow \iota v\,\,\,\text {strongly}\,\,\text {in}\,\,{\mathcal {Z}}. \end{aligned}$$

From (54), (58) and (62), we obtain

$$\begin{aligned} \int \limits _{\Omega _0\times \Delta }(a_k(x,t,Dy'_k)-a_k(x,t,Dv_k), y_k'-v_k)D\varphi \,dxdt \end{aligned}$$

63 $$\begin{aligned} \rightarrow \int \limits _{\Omega _0\times \Delta }(b(x,t)-a_\infty (x,t,\eta ), y_\infty '-v)D\varphi \,dxdt, \end{aligned}$$

and from (51), (52) and (63)

64 $$\begin{aligned} \langle \xi _k+\zeta _k, \bar{\iota }(y_k'-v_k)\varphi \rangle _{{{\mathcal {Z}}}^*\times {\mathcal {Z}}}\rightarrow \langle \xi +\zeta , \bar{\iota }(y_\infty '-v)\varphi \rangle _{{{\mathcal {Z}}}^*\times {\mathcal {Z}}} \end{aligned}$$

with $k\rightarrow \infty$. Using (61), (64), (65), $H(B)$, $H(C)$ and $(H_0)$, we pass to the limit in (60) and we obtain

65 $$\begin{aligned}&\int \limits _{\Omega _0\times \Delta }(a_k(x,t,Dy'_k)-a_k(x,t,Dv_k), Dy_k'-Dv_k)\varphi \,dxdt\nonumber \\&\qquad \rightarrow -\int \limits _{\Omega _0\times \Delta }(b(x,t)-a_\infty (x,t,\eta ), y_\infty '-v)D\varphi \,dxdt\nonumber \\&\qquad -\langle y_\infty ''-v',(y_\infty '-v_k)\varphi \rangle _{{{\mathcal {V}}}^*\times {\mathcal {V}}}-\langle \xi +\zeta ,\bar{\iota }(y_\infty '-v)\varphi \rangle _{{{\mathcal {Z}}}^*\times {\mathcal {Z}}}\nonumber \\&\qquad +\langle f_\infty +C_\infty u_\infty -v'+\text {div}\,a_\infty (x,t,Dv)-B_\infty y_\infty ,(y_\infty '-v)\varphi \rangle _{{{\mathcal {V}}}^*\times {\mathcal {V}}}\nonumber \\&\quad =\langle -y_\infty ''+\text {div}\,a_\infty (x,t,Dv)-B_\infty y_\infty -\bar{\iota }^*\xi -\bar{\iota }^*\zeta + f_\infty +C_\infty u_\infty ,(y_\infty '-v)\varphi \rangle _{{{\mathcal {V}}}^*\times {\mathcal {V}}}\nonumber \\&\qquad -\int \limits _{\Omega _0\times \Delta }(b(x,t)-a_\infty (x,t,\eta ), y_\infty '-v)D\varphi \,dxdt=I_1+I_2. \end{aligned}$$

On the other hand, taking the ${\mathcal {V}}^*_{weak}$-limit in (50), we get

66 $$\begin{aligned} y_\infty ''-\text {div}\,b(x,t)+B_\infty y_\infty +\bar{\iota }^*\xi +\bar{\iota }^*\zeta =f_\infty +C_\infty u_\infty . \end{aligned}$$

Inserting the last equality to $I_1$ and integrating by parts, we have

$$\begin{aligned} I_1&= \int \limits _{\Omega _0\times \Delta }(b(x,t)-a_\infty (x,t,\eta ),(y_\infty '-v)D\varphi )dxdt\nonumber \\&+\int \limits _{\Omega _0\times \Delta }(b(x,t)-a_\infty (x,t,\eta ),(Dy_\infty '-Dv)\varphi )dxdt. \end{aligned}$$

Hence, and from (60) we get in the limit

$$\begin{aligned} \int \limits _{\Omega _0\times \Delta }(b(x,t)-a_\infty (x,t,\eta ),(Dy_\infty '-\eta )\varphi )dxdt\ge 0. \end{aligned}$$

Denoting $g(x,t)=(b(x,t)-a_\infty (x,t,\eta ),(Dy_\infty '-\eta ))\in L^1(\Omega _0\times \Delta )$, we can take a family of mollifier kernels $\varphi _\varepsilon$ centered in $(y,s)\in \Omega _0\times \Delta$ in place of $\varphi$ and get, setting $g(x,t)=0$ outside $\Omega \times (0,T)$

$$\begin{aligned} g_\epsilon (y,s) = \int \limits _{\Omega _0\times \Delta } g(x,t)\varphi _\varepsilon (y-x,s-t)\ dxdt\ge 0. \end{aligned}$$

Since $g_\varepsilon \rightarrow g$ in $L^1(\Omega _0\times \Delta )$, for a subsequence, we know that $g_\varepsilon (x,t) \rightarrow g(x,t)$ for a.e. $(x,t)\in \Omega _0\times \Delta$. Hence, we have

$$\begin{aligned} (b(x,t)-a_\infty (x,t,\eta ),(Dy_\infty '-\eta ))=g(x,t)\ge 0 \,\,\,\text {a.e.}\,\text {in}\,\,\,\Omega _0\times \Delta ,\,\,\text {for all}\,\,\eta \in \mathbb {R}^N, \end{aligned}$$

and hence also a.e. in $Q$ for every $\eta \in \mathbb {R}^N$. Let $w\in \mathbb {R}^N$ and $\lambda >0$. Taking $\eta =Dy_\infty '+\lambda w$ we obtain

$$\begin{aligned} (b(x,t)-a_\infty (x,t,Dy_\infty '+\lambda w),w)\ge 0 \,\,\,\text {a.e.}\,\text {in}\,\,\,Q. \end{aligned}$$

Recalling that $a_\infty (x,t,\cdot )$ is continuous (by the definition of class ${\mathcal {M}}(m_0,m_1,m_2,\alpha ))$, we pass to the limit with $\lambda \rightarrow 0$ in the last inequality and obtain

$$\begin{aligned} (b(x,t)-a_\infty (x,t,Dy_\infty '),w)\ge 0 \,\,\,\text {a.e.}\,\text {in}\,\,\,Q. \end{aligned}$$

If we replace $w$ by $-w$ we deduce that $b(x,t)=a_\infty (x,t,Dy_\infty ')$ a.e.in $Q$. This together with (67) implies that $y_\infty \in {\mathcal {S}}_\infty (\mathfrak {u}_\infty )$. The proof of (43) is completed. Moreover, if ${\mathcal {S}}_\infty (\mathfrak {u}_\infty )$ is a singleton, than it follows that the whole sequence $y_k$ converges to $y_\infty$ and hence (44) follows. $\square$

## $\Gamma$-Convergence of Cost Functionals

In this section we state conditions which guarantee the suitable $\Gamma$-convergence of the cost functionals in the control problem $(CP)_{\mathcal{R}_k}$.

In the sequel we replace $H(C)$ with the following, stronger, assumption:

$\underline{H(C)^{(1)}}:$    $C_k \in \mathcal{L}(\mathcal{U}, L^q(Q))$, $k \in {\bar{\mathbb {N}}}$ are uniformly bounded and $\displaystyle C_k \ {\buildrel c \over \longrightarrow } \ C_\infty$.

We consider the following costs

67 $$\begin{aligned}&\mathcal{F}_k(\mathfrak {u},y) := \mathcal{F}_k^{(1)}(y,y') + \mathcal{F}_k^{(2)}(u) + \mathcal{F}_k^{(3)}(y_0) + \mathcal{F}_k^{(4)}(y_1),\\&\text {for}\ \ y \in {\mathcal {Y}}, \ u \in \mathcal{U}, \ y^0 \in V, y^1\in H,\nonumber \end{aligned}$$

where

68 $$\begin{aligned} \mathcal{F}_k^{(1)}(y,y') = \int \limits _Q F_k^{(1)}(x,t,y(x,t),y'(x,t)) \, dtdx, \end{aligned}$$

69 $$\begin{aligned} \mathcal{F}^{(2)}_k(u) = \int \limits _Q F_k^{(2)}(x,t,(C_k u)(x,t)) \, dtdx, \end{aligned}$$

70 $$\begin{aligned} \mathcal{F}_k^{(3)}(y^0) = \int \limits _\Omega F_k^{(3)}(x,y^0(x), Dy^0(x)) \, dx. \end{aligned}$$

71 $$\begin{aligned} \mathcal{F}_k^{(4)}(y^1) = \int \limits _\Omega F_k^{(4)}(x,y^1(x)) \, dx. \end{aligned}$$

In the following hypotheses the conditions (i), (ii), and (iii) hold uniformly with respect to $k \in {\bar{\mathbb {N}}}$.

$$\underline{H(F^{(1)})}:$$

(i) $F_k^{(1)} :Q \times {\mathbb {R}} \times {\mathbb {R}} \rightarrow {\mathbb {R}}$ is measurable in $(x,t)\in Q$, $F_k^{(1)}(x, t, 0, 0) \in L^p(Q)$;
(ii) $\displaystyle | F_k^{(1)}(x,t, z_1, w_1) - F_k^{(1)}(x,t, z_2, w_2)|$ $\displaystyle \le C_1\left( (1 + |z_1|^{p-1}) |z_1 - z_2| + (1 + |w_1|^{p-1}) |w_1 - w_2|\right)$ a.e. $(x,t)\in Q$, for all $(z_1, z_2, w_1, w_2)\in \mathbb {R}^4$ for some $C_1 > 0$;
(iii) at least one of the following two conditions holds

- $\displaystyle F_k^{(1)}(\cdot , \cdot , z, w) \ {\buildrel w-L^1(Q) \over \longrightarrow } \ F_\infty ^{(1)}(\cdot , \cdot , z, w) \ \mathrm{{for \ all}} \ (z,w) \in {\mathbb {R}^2}$ and $|F_k^{(1)}(x,t,z,w)|\le C_2$ for all $(z,w)\in \mathbb {R}^2$ a.e. $(x,t)\in Q$ with some $C_2>0$;
- $$F_k^{(1)}(x, t, z, w)\ \longrightarrow \ F_\infty ^{(1)}(x, t, z, w) \ \mathrm{{for \ all}} \ (z,w) \in {\mathbb {R}^2}$$ a.e. $$(x,t)\in Q$$;

$$\underline{H(F^{(2)})}:$$

(i) $F_k^{(2)} :Q \times {\mathbb {R}} \rightarrow {\mathbb {R}}$ is measurable in $(x,t)\in Q$, convex in $z \in {\mathbb {R}}$;
(ii) $\displaystyle |F_k^{(2)}(x,t, z)| \le C_3 (1+| z |^q)$ for all $z\in \mathbb {R}$ a.e. in $Q$ with some $C_3 > 0$;
(iii) at least one of the following two conditions holds

- $\displaystyle F_k^{(2)}(\cdot , \cdot , z) \ {\buildrel w-L^1(Q) \over \longrightarrow } \ F_\infty ^{(2)}(\cdot , \cdot , z) \ \mathrm{{for \ all}} \ z \in {\mathbb {R}}$ and $|F_k^{(2)}(x,t,z)|\le C_4$ for all $z\in \mathbb {R}$ a.e. $(x,t)\in Q$ with some $C_4>0$;
- $$F_k^{(2)}(x, t, z) \ \longrightarrow \ F_\infty ^{(2)}(x, t, z) \ \mathrm{{for \ all}} \ z \in {\mathbb {R}}$$ and a.e. $$(x,t)\in Q$$;

$$\underline{H(F^{(3)})}:$$

(i) $F_k^{(3)} :\Omega \times {\mathbb {R}}^{N+1} \rightarrow {\mathbb {R}}$ is measurable in $x \in \Omega$, convex in $z \in {\mathbb {R}}^{N+1}$;
(ii) $\displaystyle |F_k^{(3)}(x, z)| \le C_5 (1+|z|^p)$ for all $z\in \mathbb {R}^{N+1}$ a.e. in $\Omega$ with some $C_5 > 0$;
(iii) at least one of the following two conditions holds

- $\displaystyle F_k^{(3)}(\cdot , \cdot , z) \ {\buildrel w-L^1(Q) \over \longrightarrow } \ F_\infty ^{(3)}(\cdot , \cdot , z) \ \mathrm{{for \ all}} \ z \in {\mathbb {R}^{N+1}}$ and $|F_k^{(3)}(x,t,z)|\le C_6$ for all $z\in \mathbb {R}^{N+1}$ a.e. $(x,t)\in Q$ with some $C_6>0$;
- $$F_k^{(3)}(x, t, z) \ \longrightarrow \ F_\infty ^{(3)}(x, t, z) \ \mathrm{{for \ all}} \ z \in {\mathbb {R}^{N+1}}$$ and a.e. $$(x,t)\in Q$$;

$$\underline{H(F^{(4)})}:$$

(i) $F_k^{(4)} :\Omega \times {\mathbb {R}} \rightarrow {\mathbb {R}}$ is measurable in $x \in \Omega$, convex in $z \in {\mathbb {R}}$;
(ii) $\displaystyle F_k^{(4)}(x, z) \le C_7 (1+|z|^2)$ for all $z\in \mathbb {R}$ a.e. in $\Omega$ with some $C_7 > 0$;
(iii) at least one of the following two conditions holds

- $\displaystyle F_k^{(4)}(\cdot , \cdot , z) \ {\buildrel w-L^1(Q) \over \longrightarrow } \ F_\infty ^{(4)}(\cdot , \cdot , z) \ \mathrm{{for \ all}} \ z \in {\mathbb {R}}$ and $|F_k^{(4)}(x,t,z)|\le C_8$ for all $z\in \mathbb {R}$ a.e. $(x,t)\in Q$ with some $C_8>0$;
- $$F_k^{(4)}(x, t, z) \ \longrightarrow \ F_\infty ^{(4)}(x, t, z) \ \mathrm{{for \ all}} \ z \in {\mathbb {R}}$$ and a.e. $$(x,t)\in Q$$;

### **Proposition 22**

For every fixed $k \in {\bar{\mathbb {N}}}$, under the regularity assumptions (i), (ii) of $H(F^{(1)})$, $H(F^{(2)})$, $H(F^{(3)})$ and $H(F^{(4)})$, respectively, we have

(j) $\mathcal{F}_k^{(1)}(\cdot )$ is $s-W^{1,p}(0,T;L^p(\Omega ))$ so also sequentially $\Xi -{\mathcal {Y}}$ continuous,
(jj) $\mathcal{F}_k^{(2)}(\cdot )$ is $s-{\mathcal {U}}$ continuous,
(jjj) $\mathcal{F}_k^{(3)}(\cdot )$ is $s-V$ continuous,
(jv) $\mathcal{F}_k^{(4)}(\cdot )$ is $s-H$ continuous.

Also, under the convergence conditions (iii) of $H(F^{(1)})$, $H(F^{(2)})$, $H(F^{(3)})$ and $H(F^{(4)})$, respectively, we have


and for functional $\displaystyle \mathcal{F}_k(u,y^0,y^1,y)$ defined by (68), we obtain $\,\,\, \displaystyle \mathcal{F}_\infty (u,y^0,y^1,y) = \quad \displaystyle \Gamma _{seq} \left( s-{\mathcal {U}}^{-}, s-V^{-},s-H^{-},\Xi -{\mathcal {Y}} \right) \lim _{k\rightarrow \infty } \mathcal{F}_k(u, y^0,y^1,y)$.

### *Proof*

For the proof of $(j)$ assume that $y_n\rightarrow y$ strongly in $W^{1,p}(0,T;L^p(\Omega ))$. This means that $y_n\rightarrow y$ strongly in $L^p(Q)$ and $y_n'\rightarrow y'$ strongly in $L^p(Q)$. From $H(F^{(1)})(ii)$, we obtain

72 $$\begin{aligned}&|{\mathcal {F}}^{(1)}_k(y)-{\mathcal {F}}^{(1)}_k(y_n)|\nonumber \\&\quad \le \int \limits _{Q}| F_k^{(1)}(x,t, y(x,t), y'(x,t)) - F_k^{(1)}(x,t, y_n(x,t),y_n'(x,t))|\ dxdt\nonumber \\&\quad \le C_1\left( \int \limits _Q 2^{q-1}(1 + |y(x,t)|^p) \ dxdt\right) ^{\frac{1}{q}}\Vert y_n-y\Vert _{L^p(Q)} \nonumber \\&\qquad + C_1\left( \int \limits _Q 2^{q-1}(1 + |y'(x,t)|^p) \ dxdt\right) ^{\frac{1}{q}}\Vert y'_n-y'\Vert _{L^p(Q)}, \end{aligned}$$

and the assertion follows. The continuity of ${\mathcal {F}}^{(i)}_k$ for $i=2,3,4$ follows from the fact that convex and locally bounded functions are continuous and from Carathéodory Continuity Theorem (see Example 1.22 in [12]).

To prove the continuous convergence of ${\mathcal {F}}_{k}^{(1)}$, assume that $y_k\rightarrow y$ in $\Xi$. We have

$$\begin{aligned} |{\mathcal {F}}_{k}^{(1)}(y_k) - {\mathcal {F}}_{\infty }^{(1)}(y)| \le |{\mathcal {F}}_{k}^{(1)}(y_k) - {\mathcal {F}}_{k}^{(1)}(y)| + |{\mathcal {F}}_{k}^{(1)}(y) - {\mathcal {F}}_{\infty }^{(1)}(y)|. \end{aligned}$$

The first term on the right hand side tends to zero uniformly in $k$ by (73). To prove the convergence of the second term, we proceed separately for the cases $H(F^{(1)})\mathrm{(iiia)}$ and $H(F^{(1)})\mathrm{(iiib)}$. For the proof in the first case choose $y\in {\mathcal {Y}}$ to get

$$\begin{aligned} | {\mathcal {F}}_k^{(1)}(y) - {\mathcal {F}}_\infty ^{(1)}(y) |&\le \int \limits _{Q} | F_k^{(1)} (x,t,y(x,t),y'(x,t))\nonumber \\&- F_\infty ^{(1)} (x,t,y(x,t),y'(x,t))| \ dtdx. \end{aligned}$$

By the Luzin theorem we know that for every $\varepsilon > 0$ we can find a compact set $K\subset Q$ such that $y(x,t)$ and $y'(x,t)$ are continuous on $K$ and $\mu _{N+1}(Q\setminus K)<\varepsilon$ (where $\mu _{N+1}$ stands for $N+1$ dimensional Lebesgue measure). Hence, we have

$$\begin{aligned}&| {\mathcal {F}}_k^{(1)}(y) - {\mathcal {F}}_\infty ^{(1)}(y) | \le 2C_2 \varepsilon \nonumber \\&\quad + \int \limits _{K} | F_k^{(1)} (x,t,y(x,t),y'(x,t)) - F_\infty ^{(1)} (x,t,y(x,t),y'(x,t))| \ dtdx. \end{aligned}$$

Now the fact that the last term in above inequality can be made arbitrarily small follows from the convergence $\displaystyle F_k^{(1)}(\cdot , \cdot , z, w)\ {\buildrel w-L^1(Q) \over \longrightarrow } \ F_\infty ^{(1)}(\cdot , \cdot , z, w) \ \mathrm{{for \ all}} \ (z,w) \in {\mathbb {R}^2}$ in the similar way as the convergence (13) in Lemma 4.1 in [17]. The proof in the case $H(F^{(1)})\mathrm{(iiib)}$ follows by a direct application of the Lebesgue dominated convergence theorem. For the proof of $\Gamma$-convergence of ${\mathcal {F}}^{(2)}_k$, ${\mathcal {F}}^{(3)}_k$, and ${\mathcal {F}}^{(4)}_k$ observe that these functions are convex and locally equibounded. Hence, by Proposition 5.12 in [12], the $\Gamma$-convergence is equivalent to the pointwise convergence. The pointwise convergence follows from (iii) in the same way as for ${\mathcal {F}}^{(1)}$. This completes the proof. $\square$

Now, the main result on the sensitivity of optimal control problems follows from Proposition 5, Theorem 20, Proposition 22 and the direct method for the existence part.

### **Theorem 23**

Under the hypotheses $H(A)$, $H(B)$, $H(J^1)$, $H(J^2)$, $H(C)^{(1)}$, $(H_0)$ and $(H_1)$ for $(P)_k$, we admit the hypotheses $H(F^{(j)})$, $j = 1$, $2$, $3$, $4$ for cost functionals $\mathcal{F}_k(u, y^0, y^1, y)$ given by (68). Moreover, let the space of admissible controls $\mathfrak {U}$ be compact in ${\mathcal {U}}\times V\times H$ (alternatively we can assume that sublevel sets of the functionals ${\mathcal {F}}^{(2,3,4)}_k:\mathfrak {U}\rightarrow \mathbb {R}$ defined as ${\mathcal {F}}^{(2,3,4)}_k(\mathfrak {u})=\mathcal{F}_k^{(2)}(u) + \mathcal{F}_k^{(3)}(y_0) + \mathcal{F}_k^{(4)}(y_1)$ are compact). Then

(i) For every $k \in {\bar{\mathbb {N}}}$ the problem $(CP)_{\mathcal{R}_k}$ has at least one optimal solution $y_k^* \in {\mathcal {S}}_k(u_k^*, y_k^{0*},y_k^{1*})$, $m_k := \mathcal{F}_k (u_k^*, y_k^{0*},y_k^{1*},y_k^*)$ being its minimal value.
(ii) If the limit (original) problem $(CP)_{\mathcal{R}_\infty }$ has the “uniqueness of solution property”, i.e., for all $u \in \mathcal{U}$, $\mathcal{S}_{\infty }(u) = \{ y_\infty (u) \}$ (see Theorem 19), then the sequence $(u_k^*, y_k^{0*}, y_k^{1*}, y_k^*)$ has a cluster point and, moreover, every cluster point to this sequence is an optimal solution to the problem $(CP)_{\mathcal{R}_\infty }$, i.e.,
  $$\begin{aligned}&(u_{k_n}^*, y_{k_n}^{0*}, y_{k_n}^{1*},y_{k_n}^*) {\buildrel (s-{\mathcal {U}})\times (s-V)\times (s-H)\times (\Xi -{\mathcal {Y}}) \over \longrightarrow } (u_\infty ^*, y_\infty ^{0*}, y_\infty ^{1*},y_\infty ^*) \ \mathrm{{and}} \\&\ y_\infty ^* \in \mathcal{S}_\infty ^*( u_\infty ^*, y_\infty ^{0*}, y_\infty ^{1*}) . \end{aligned}$$
(iii) We also have $m_k \rightarrow m_\infty \ \mathrm{as} \ k \rightarrow \infty .$

## Examples

In this section we give examples of particular operators and functionals, for which the results of the paper are applicable. Let $p=2$ and $V=H^1_0(\Omega )$. We provide examples for the following hypotheses

*H(A)*: Note that the operators $A_k$, $k\in \bar{\mathbb {N}}$, are given in the explicit form in the assumption $H(A)$. Svanstedt (see Theorem 3.1 in [49]) proves that any sequence $\{a_k\}_{k=1}^\infty$ such that $a_k\in {\mathcal {M}}$ has a subsequence such that $a_k \ {\buildrel PG \over \longrightarrow } \ a_\infty$ with some $a_\infty \in {\mathcal {M}}$.
*H(B)*: The condition $H(B)\mathrm{(ii)}$ holds for the operators defined by
  $$\begin{aligned}&\langle B_ky,v \rangle =\int \limits _{\Omega }g_k(x)y(x)v(x)+(\nabla y(x)\cdot h_k(x))v(x)dx,\nonumber \\&\text {for}\,\,\,y, v\in \mathcal{V}, k\in \bar{\mathbb {N}}, \end{aligned}$$
  where $g_k\rightarrow g_{\infty }$ in $L^{\infty }(\Omega )$ and $h_k\rightarrow h_{\infty }$ in $W^{1,\infty }(\Omega ;\mathbb {R}^N)$. Moreover, if $h_k\equiv 0$ and $g_k\ge 0$, then also the condition $H(B)$(i) holds. Note that the second order operator (for example the Laplace operator) is not allowed here.
*H(J)*: Let $j:\mathbb {R}\rightarrow \mathbb {R}$ be a locally Lipschitz function such that $\xi r\ge 0$ for all $r\in \mathbb {R}$ and $\xi \in \partial j(r)$, and $|\xi |\le C(1+|r|^{p-1})$ for all $r\in \mathbb {R}$ and $\xi \in \partial j(r)$. For example, we can put $j(r)=|r|$. We define $Z=L^p(\Omega )$ and $J:Z\rightarrow \mathbb {R}$ as $J(z)=\int _{\Omega } j(z(x))\, dx$. We take a sequence of mollifier kernels $\varrho _k=k\varrho (kx)$, where $\varrho \in C^\infty (\mathbb {R})$, $supp(\varrho )\subset [-1,1]$ and $\varrho (x)\ge 0$ for all $x\in \mathbb {R}$. We define $j_n(r)=\int _{\mathbb {R}}\varrho _n(s)j(r-s)\, ds$, and the associated functional given by $J_n(z)=\int _{\Omega }j_n(z(x))\, dx$. A straightforward computation proves that $J$ and $J_n$ satisfy $H(J^1)$(i)-(ii) and $H(J^2)$(i) uniformly in $n\in \mathbb {N}$. To show that $H(J^1)$(iii) and $H(J^2)$(ii) hold, we observe that by the computation analogous to the one given in the proof of Theorem 3.1 in [30], we have
  $$\begin{aligned} K-\limsup _{k\rightarrow \infty } Gr\, j_k' \subset Gr\, \partial j. \end{aligned}$$
  Using Theorem 3 in [52], we recover $H(J^1)$(iii) and $H(J^2)$(ii). Note that Theorem 1 of Zolezzi in [52] formulates abstract conditions which assure that $H(J^1)$(iii) and $H(J^2)$(ii) hold.
*H(C)*: Note that $H(C)$ holds provided $C_ku\rightarrow C_{\infty }u$ strongly in $\mathcal{V}^*$ for all $u\in \mathcal{U}$ and $C_k\in \mathcal{L}(\mathcal{U}, \mathcal{V}^*)$ are uniformly bounded. Let $\mathcal{U}=\mathbb {R}^M$, $M\in \mathbb {N}$ and for $u\in \mathcal{U}$, we denote $u=(u_1,\ldots ,u_M)$. Define $C_ku=\sum _{i=1}^Mu_iw_i^k$, where $w_i^k\in \mathcal{V}^*$ for $i=1,\ldots , M$ and $k\in \bar{\mathbb {N}}$ are such that $w_i^k\rightarrow w_i^{\infty }$ strongly in $\mathcal{V}^*$ for all $i=1,\ldots ,M$. Then the required convergence holds.
*H(F)*: We give example of the sequence of functionals $F_k^{(1)}$, with $k\in \overline{\mathbb {N}}$, that satisfy $H(F^{(1)})$. Note that corresponding examples that satisfy $H(F^{(2)})-H(F^{(4)})$ can be constructed analogously. Let $z_k\in L^p(Q)$ for $k\in \overline{\mathbb {N}}$ be such that $z_k(x,t)\rightarrow z_\infty (x,t)$ for a.e. $(x,t)\in Q$ and $w_k\in L^p(Q)$ for $k\in \overline{\mathbb {N}}$ be such that $w_k(x,t)\rightarrow w_\infty (x,t)$ for a.e. $(x,t)\in Q$. Define $F^{(1)}_k(x,t,z,w)=|z-z_k(x,t)|+|w-w_k(x,t)|$. Then $H(F^{(1)})$ (i)–(ii) and (iiib) obviously hold.

## References

[1] Acquistapace, P., Briani, A.: $$\Gamma $$-convergence for infinite dimensional optimal control problems. Evolution equations, semigroup and functional analysis (2000), 1–25, Progress in Nonlinear Differential Equations and Their Applications. 50, Birkhäuser, Basel (2002)

[2] Arada N, Raymond JP (1999). Stability analysis of relaxed Dirichlet boundary control problems. Control Cybern..

[3] Attouch H (1984). Variational Convergence for Functions and Operators.

[4] Aubin JP, Frankowska H (1990). Set-Valued Analysis.

[5] Bartosz K, Kalita P (2012). Optimal control for a class of dynamic viscoelastic contact problems with adhesion. Dyn. Syst. Appl..

[6] Briani A (2000). Convergence of Hamilton–Jacobi equations for sequences of optimal control problems. Commun. Appl. Anal..

[7] Buttazzo G, Dal Maso G (1982). $$\Gamma $$-convergence and optimal control problems. J. Optim. Theory Appl..

[8] Buttazzo G, Freddi L (1993). Sequences of optimal control problems with measures as controls. Adv. Math. Sci. Appl..

[9] Buttazzo G, Freddi L (1995). Optimal control problems with weakly converging input operators. Discret. Contin. Dyn. Syst..

[10] Clarke FH (1983). Optimization and Nonsmooth Analysis.

[11] Colombini F, Spagnolo S (1977). Sur la convergence de solutions d’equations paraboliques. J. Math. Pures Appl..

[12] Dal Maso G (1993). An Introduction to $$\Gamma $$-Convergence.

[13] De Giorgi E, Spagnolo S (1973). Sulla convergenza degli integrali dell’energia per operatori ellitici del secondo ordine. Boll. Della Unione Mat. Ital..

[14] De Giorgi E, Franzoni T (1975). Su un tipo di convergenza variazionale. Atti Accad. Naz Lincei Rend. Cl. Sci. Fis. Natur..

[15] Denkowski Z, Migórski S (1987). Control problems for parabolic and hyperbolic equations via the theory of *G*- and $$\Gamma $$-convergence. Annal. Mate. Pura Appl..

[16] Denkowski Z, Mortola S (1993). Asymptotic behaviour of optimal solutions to control problems for systems described by differential inclusions corresponding to partial differential equations. J. Optim. Theory Appl..

[17] Denkowski Z, Staicu V (1994). Asymptotic behaviour of the minima to a class of optimization problems for differential inclusions. J. Optim. Theory Appl..

[18] Denkowski Z, Migórski S (1998). Optimal shape design problems for a class of systems described by hemivariational inequalities. J. Glob. Optim..

[19] Denkowski Z, Migórski S, Schmidt WH, Heier K, Bittner L, Bulirsch R (1998). Optimal shape design for elliptic hemivariational inequalities in nonlinear elasticity. International Series of Numerical Mathematics.

[20] Denkowski Z (2000). Existence and relaxation problems in optimal shape design. Topol. Methods Nonlinear Anal..

[21] Denkowski, Z.: A survey on optimal shape design problems for systems described by PDE’s and hemivariational inequalities. From Convexity to Nonconvexity, Nonconvex Optimization and its Applications, pp. 51–66. Kluwer Academic, Boston (2001)

[22] Denkowski Z (2002). Control problems for systems described by hemivariational inequalities. Control Cybern..

[23] Denkowski Z, Migórski S, Papageorgiou NS (2003). An Introduction to Nonlinear Analysis: Theory.

[24] Denkowski Z, Migórski S, Papageorgiou NS (2003). An Introduction to Nonlinear Analysis: Applications.

[25] Denkowski Z, Migórski S (2004). Sensitivity of optimal solutions to control problems for systems described by hemivariational inequalities. Control Cybern..

[26] Duvaut G, Lions JL (1976). Inequalities in Mechanics and Physics.

[27] Freddi L (2000). Optimal control problems with weakly converging input operators in a nonreflexive framework. Port. Math..

[28] Gasiński L (1998). Optimal shape design problems for a class of systems described by parabolic hemivariational inequalities. J. Glob.Optim..

[29] Haslinger J, Panagiotopoulos PD (1995). Optimal control of systems governed by hemivariational inequalities. Existence and approximation result. Nonlinear Anal. Theory Methods Appl..

[30] Kalita, P., Łukaszewicz, G.: Attractors for Navier-Stokes flows with multivalued and nonmonotone subdifferential boundary conditions. Nonlinear Anal. Real World Appl. **19**, 75–88 (2014)

[31] Migórski S (1992). Asymptotic behaviour of optimal solutions in control problems for elliptic equations. Riv. Mat. Pura Appl..

[32] Migórski S (1992). On asymptotic limits of control problems with parabolic and hyperbolic equations. Riv. Mate. Pura Appl..

[33] Migórski S (1995). Sensitivity analysis of distributed optimal control problems for nonlinear parabolic equations. J. Optim. Theory Appl..

[34] Migórski S (1999). Sensitivity analysis of inverse problems with applications to nonlinear systems. Dyn. Syst. Appl..

[35] Migórski S, Ochal A (2000). Inverse coefficient problem for elliptic hemivariational inequality. Nonsmooth/Nonconvex Mechanics: Modeling, Analysis and Numerical Methods.

[36] Migórski S, Ochal A (2000). Optimal control of parabolic hemivariational inequalities. J. Glob. Optim..

[37] Migórski S, Misra JC (2003). Modeling, analysis and optimal control of systems governed by hemivariational inequalities. Industrial Mathematics. Dedicated to commemorate the Golden Jubilee of Indian Institute of Technology, Kharagpur, India, invited paper.

[38] Migórski S (2005). Boundary hemivariational inequalities of hyperbolic type and applications. J. Glob. Optim..

[39] Naniewicz Z, Panagiotopoulos PD (1995). Mathematical Theory of Hemivariational Inequalities and Applications.

[40] Nguetseng G, Nnang H, Svanstedt N (2010). G-convergence and homogenization of monotone damped hyperbolic equations. Banach J. Math. Anal..

[41] Nguetseng G, Nnang H, Svanstedt N (2011). Deterministic homogenization of quasilinear damped hyperbolic equations. Acta Math. Sci..

[42] Nnang H (2012). Deterministic homogenization of weakly damped nonlinear hyperbolic–parabolic equations. Nonlinear Differ. Equ. Appl..

[43] Ochal A (2000). Domain identification problem for elliptic hemivariational inequality. Topol. Methods Nonlinear Anal..

[44] Panagiotopoulos PD (1985). Nonconvex problems of semipermeable media and related topics. Z. Angew. Math. Mech..

[45] Panagiotopoulos PD (1985). Inequality Problems in Mechanics and Applications. Convex and Nonconvex Energy Functions.

[46] Panagiotopoulos PD (1993). Hemivariational Inequalities. Applications in Mechanics and Engineering.

[47] Papageorgiou NS, Papalini F, Renzacci F (1999). Existence of solutions and periodic solutions for nonlinear evolution inclusions. Rend. Circ. Mate. Palermo.

[48] Spagnolo, S.: Convergence in energy for elliptic operators. In: Proceedings of the Third Symposium on the Numerical Solution of Solution of Partial Differential Equations, New York, pp. 469–498 (1975)

[49] Svanstedt N (1999). *G*-convergence of parabolic operators. Nonlinear Anal..

[50] Svanstedt N (2007). Convergence of quasi-linear hyperbolic equations. J. Hyperbolic Differ. Equ..

[51] Zeidler E (1990). Nonlinear Functional Analysis and Applications, II A/B.

[52] Zolezzi T (1994). Convergence of generalized gradients. Set Valued Anal..

